# Cerebellar Development and the Burden of Prematurity

**DOI:** 10.1007/s12311-025-01790-6

**Published:** 2025-01-30

**Authors:** Tobias Muehlbacher, Jeroen Dudink, Sylke J. Steggerda

**Affiliations:** 1 Department of Neonatology, Newborn Research Zurich, University Hospital Zurich, Zurich, Switzerland; 2 Department of Neonatology, Wilhelmina Children’s Hospital, University Medical Center Utrecht, Utrecht, the Netherlands; 3 Department of Pediatrics, Division of Neonatology, Willem-Alexander Children’s Hospital, Leiden University Medical Center, Utrecht, the Netherlands

**Keywords:** Cerebellum, Cerebellar disease, Preterm infant, Neonate

## Abstract

The role of the cerebellum in the neurodevelopmental outcomes of preterm infants has often been neglected. However, accumulating evidence indicates that normal cerebellar development is disrupted by prematurity-associated complications causing cerebellar injury and by prematurity itself. This hampers not only the normal development of motor skills and gait, but also cognitive, language, and behavioral development, collectively referred to as "developmental cognitive affective syndrome." In this comprehensive narrative review, we provide the results of an extensive literature search in PubMed and Embase to summarize recent evidence on altered cerebellar development in premature infants, focusing on neuroimaging findings, its causative factors and its impact on long-term neurodevelopmental outcomes.

## Introduction

The cerebellum is interconnected with multiple subcortical and cortical regions: the spinal cord, limbic and vestibular system and as part of large brain-wide neuronal networks integrating information from the different systems [1–3]. Thus, the cerebellum is not only involved in motor function but also in cognition, language, visuospatial integration, attention, executive function and behavior [2–5]. Schmahmann and Sherman coined the description of the “cerebellar cognitive affective syndrome” in adults with cerebellar lesions [6]. To adapt this entity to the symptoms and long-term impairments of infants with prematurity-related cerebellar injury Brossard-Racine et al. complemented the name as “developmental cerebellar cognitive affective syndrome” [7]. It is important to note that these impairments are not only a result from direct lesions but also but also from the disruption of normal cerebellar developmental programming following preterm birth [8].

**Fig. 1:**
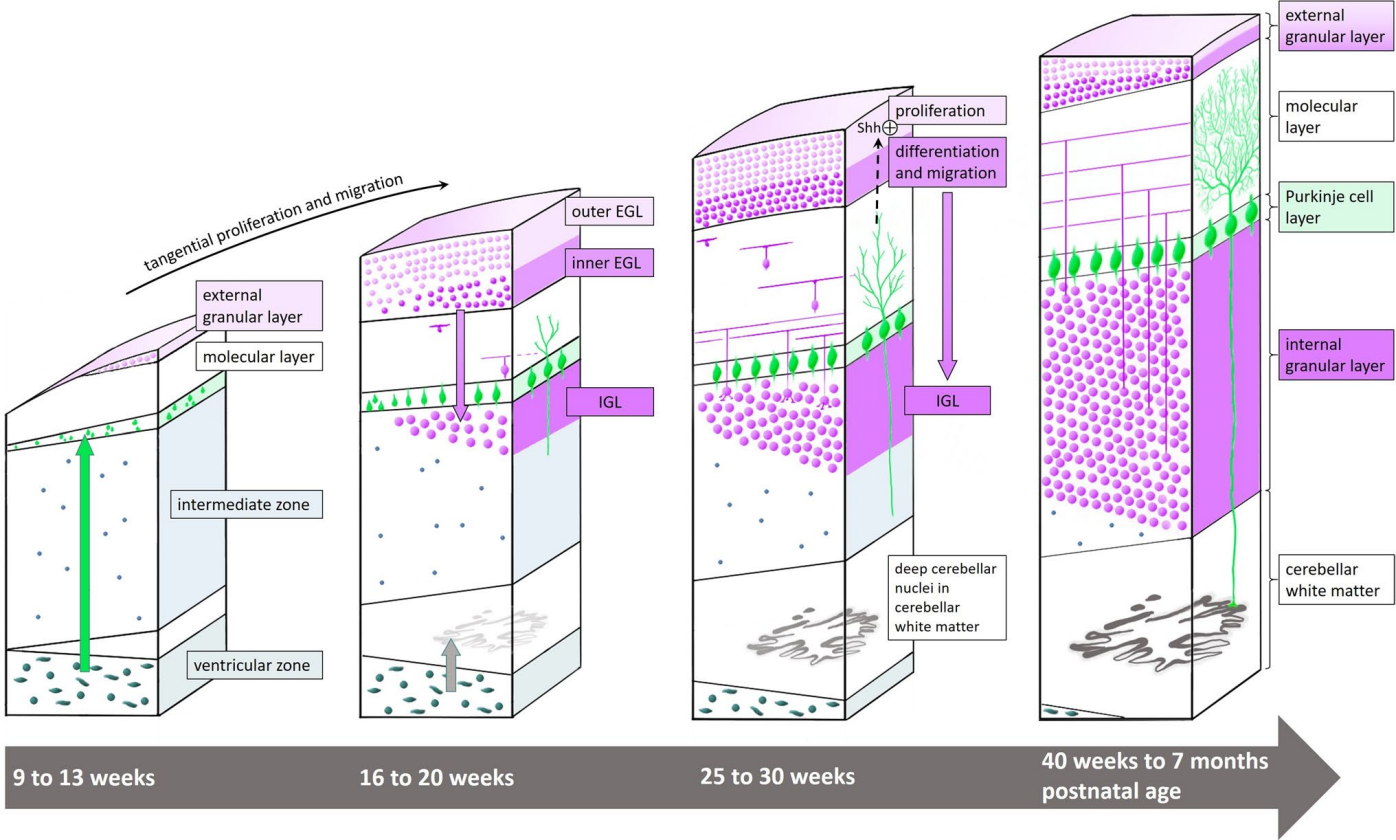
Development of the cerebellar cortex. Two proliferative zones are present in the developing cerebellum. The first is the dorsolateral subventricular zone of the rhombic lips from where excitatory granule precursor cells migrate from the 5th week of gestation and proliferate tangentially to form the external granular layer (EGL) above the developing molecular layer. The second is the ventricular zone from where gamma-aminobutyric acid (GABA)ergic inhibitory cells including the Purkinje cells (PC) originate and migrate through the intermediate zone to form the PC layer and inhibitory interneurons within the molecular layer as well as the cerebellar nuclei within the cerebellar white matter [9, 15]. At week 16, the PC layer is still organized in multiple layers before becoming the characteristic PC monolayer at 28 weeks. The EGL is a transient developmental layer reaching a peak thickness with six to nine cell layers around 25 weeks of gestation. The outer cell layers of the EGL (adjacent to the cerebrospinal fluid) proliferate under the secretion of Sonic Hedge Hog (Shh) from the growing dendritic tree of the PC, while GCP in the inner EGL start to differentiate and migrate through the molecular layer. During the migration into the initially paucicellular internal granular layer (IGL), the T-shaped axons grow perpendicular as so-called parallel fibers forming en-passant synapses with the dendritic tree of the PC. With the huge numbers of migrating GCP and their parallel fibers, the molecular layer and IGL grow from the neonatal period and during the first year of life, while the EGL gradually fades and vanishes during this period. Illustration inspired by Volpe [9].

The cerebellum starts to develop from the first month of gestation with the most rapid growth between 24 and 40 weeks, when there is a fivefold increase in volume and a 30- to 50-fold increase in cerebellar surface. During this period, which corresponds with the period of preterm birth, no other brain structure grows as fast as the cerebellum [9, 10]. After term equivalent age (TEA) cerebellar growth continues at a slower pace until at least 2 years, and even slower to 4 years of age [11] and adolescence [12]. The normal cerebellar development is presented in Fig. 1, illustrating proliferation and migration of the different cell types during this long-lasting developmental period. In the mature brain, the cerebellum contains 80 percent of all neurons, and 95 of these are granule cells (GC) [13, 14].

**Fig. 2:**
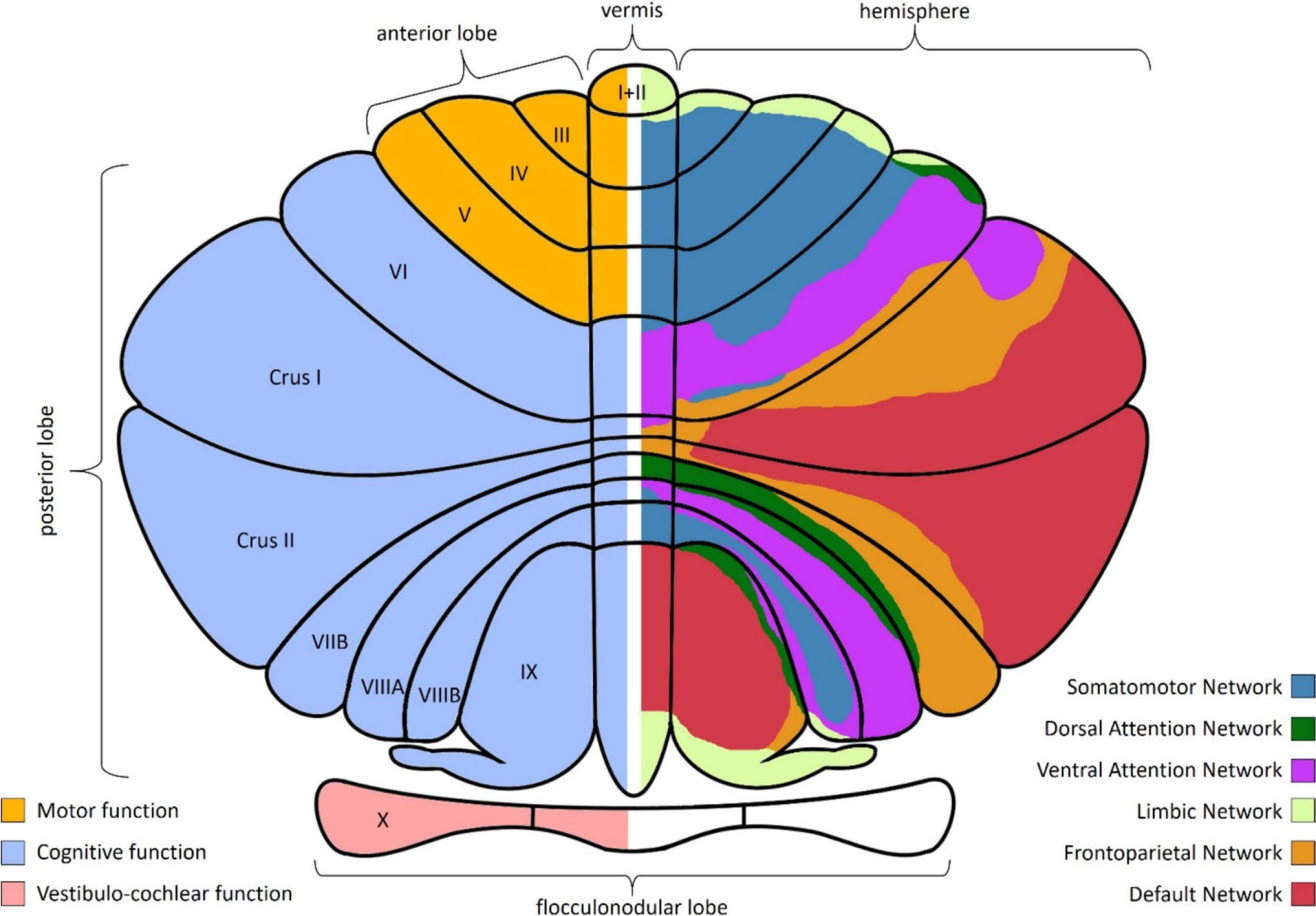
Illustration of cerebellar functional topography. Left part of the illustration: The anterior lobe (orange) consists of lobules I-V and has primarily motor function. The posterior lobe (light-blue) is the largest part of the cerebellum consisting of lobules VI to XI and is involved in cognitive function. The oldest part of the cerebellum is the floccularnodular lobe (lobule X) with direct connections to the vestibular nuclei. The right part of the illustration is a schematic of large different cerebral-cerebellar networks. Lobules III-VI and VIIIB are part of the somatomotor networks, while the rest of the cerebellum is connected with association cortices for higher cognitive function. Intriguingly, there seems to be no connection to the primary visual cortex networks [2].

Development of the cerebellum follows complex programming resulting in a highly organized microstructure [9] and a functional organization of cortico-cerebellar connections, e.g., projections from the somato-motor cortex via the pontine relay nuclei to distinct regions of the contralateral cerebellar cortex and feedback-projections which pass via the cerebellar nuclei to the contralateral thalamus and close the loop back to the cerebral cortex [16, 17]. This in turn results in a specific functional cerebellar topography (Fig. 2) [18, 19].

**Fig. 3:**
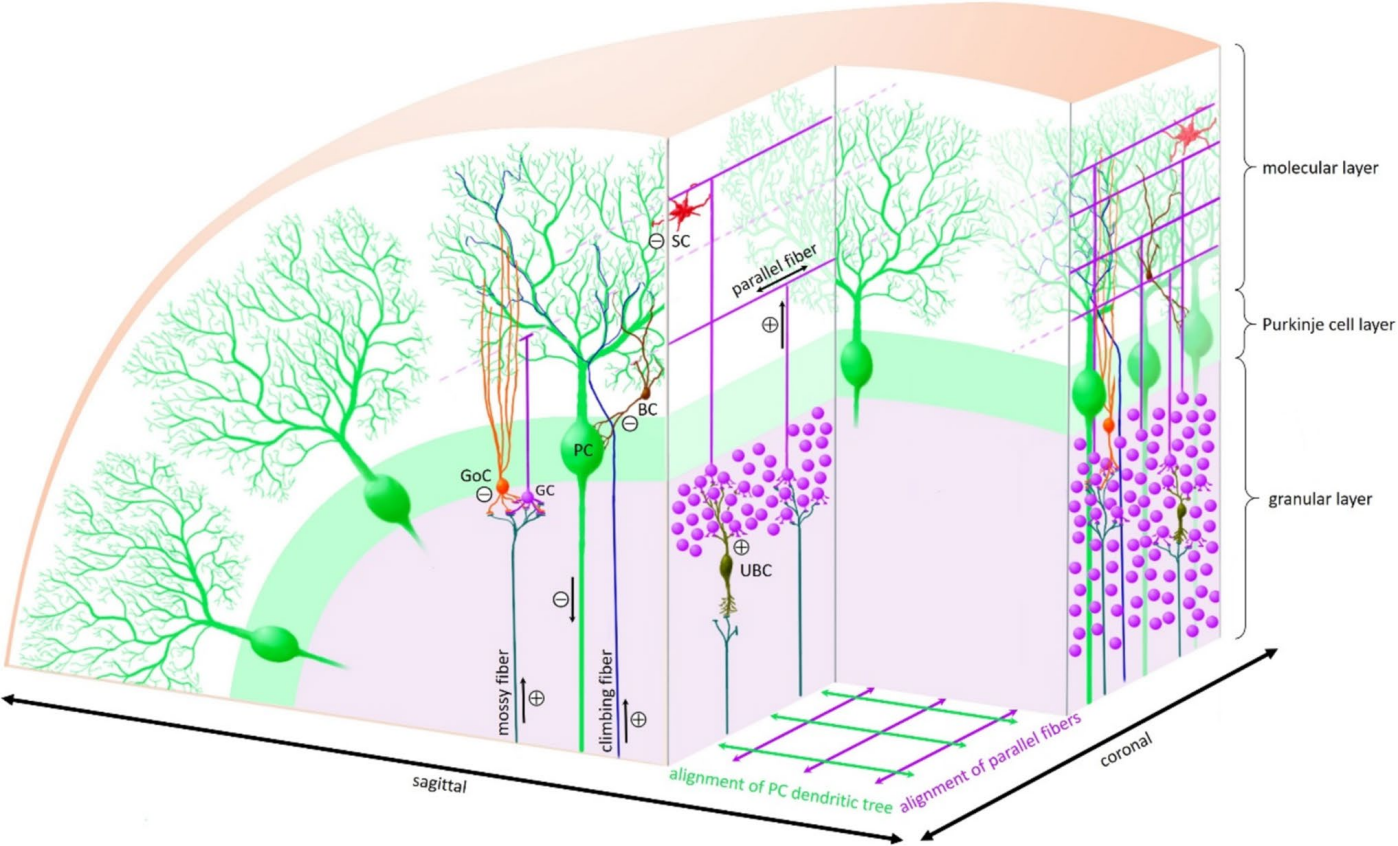
Mature cerebellar cortex and circuitry. The cerebellar cortex contains of three layers: the deepest cell-dense granular layer, the second mono-cell layer contains Purkinje cells (PC) and Bergmann glia cells and the molecular layer containing only few inhibitory interneurons, e.g. stellate cells (SC) and basket cells (BC) [9, 15]. Information from the cerebral cortex is relayed in pontine nuclei and reach excitatory granule cells (GC) and unipolar brush cells (UBC) as well as inhibitory Golgi-cells (GoC) via mossy fibers. Further afferent signaling from the spinal cord and vestibular system arrive via climbing fibers coming from the inferior olive nucleus, which interact directly with the dendritic tree of the PC. The latter are arranged strictly in a sagittal plane. Perpendicular to these in a coronal plane are the T-shaped axons (parallel fibers) of the GC which form “en-passant” synapses with the dendritic trees of multiple PC [15]. The only efferent signals from the cerebellar cortex are transferred via the axons of the PC to the cerebellar nuclei in the cerebellar white matter (from lateral to ventral the dentate, emboliform, globose and fastigial nuclei), which relay the information mainly via the thalamus to the cerebral cortex or to lesser extend to red nucleus or other structures in the brainstem [15, 20]. Afferent fibers from the cerebral cortex are relayed in the pontine nuclei (mossy fibers) and arrive in the contralateral cerebellum via the middle cerebellar peduncle [17, 20]. The inferior cerebellar peduncle contains afferent projections from the inferior olive nucleus (climbing fibers) and both afferent and efferent fibers from and to the spinal cord and vestibular system [16, 20].

Figure 3 illustrates the mature cerebellar cortex with its characteristic layering, afferent and efferent information flow, and the involved cell types.

Because the cerebellum develops over a long period, with rapid growth and changes in structure and connectivity during the third trimester, it is vulnerable to injury and altered development. This is especially true after premature birth, when stressors in the neonatal intensive care unit (NICU) environment can affect its development [8].

### Aim of the Study

The overall aim of this study was to create a comprehensive review on normal and disrupted cerebellar development in preterm infants. Therefore, we organized this review in the following sections:

- imaging cerebellar development and injury with a set of exemplary images of normal and abnormal findings,
- pathophysiological mechanisms leading to the disruption of the normal development with recent evidence from animal and human studies,
- comparing prenatal and postnatal cerebellar growth in the neonatal period to highlight the burden of prematurity and
- the long-term effect of an altered cerebellar development on neurodevelopmental outcome.

## Method and Results

For this narrative review we conducted an extensive literature search up to July 2024 in PubMed and Embase using the terms ((cerebellar injury) AND ((neonate) OR (preterm))); ((cerebellar hypoplasia) AND ((neonate) OR (preterm))) resulting in 1058 studies after removing 240 duplicates. After abstract and full text screening and adding studies from cross-references and peer review process, 111 original studies or meta-analyses have been included in this study.

### Imaging Cerebellar Development and Injury

**Fig. 4:**
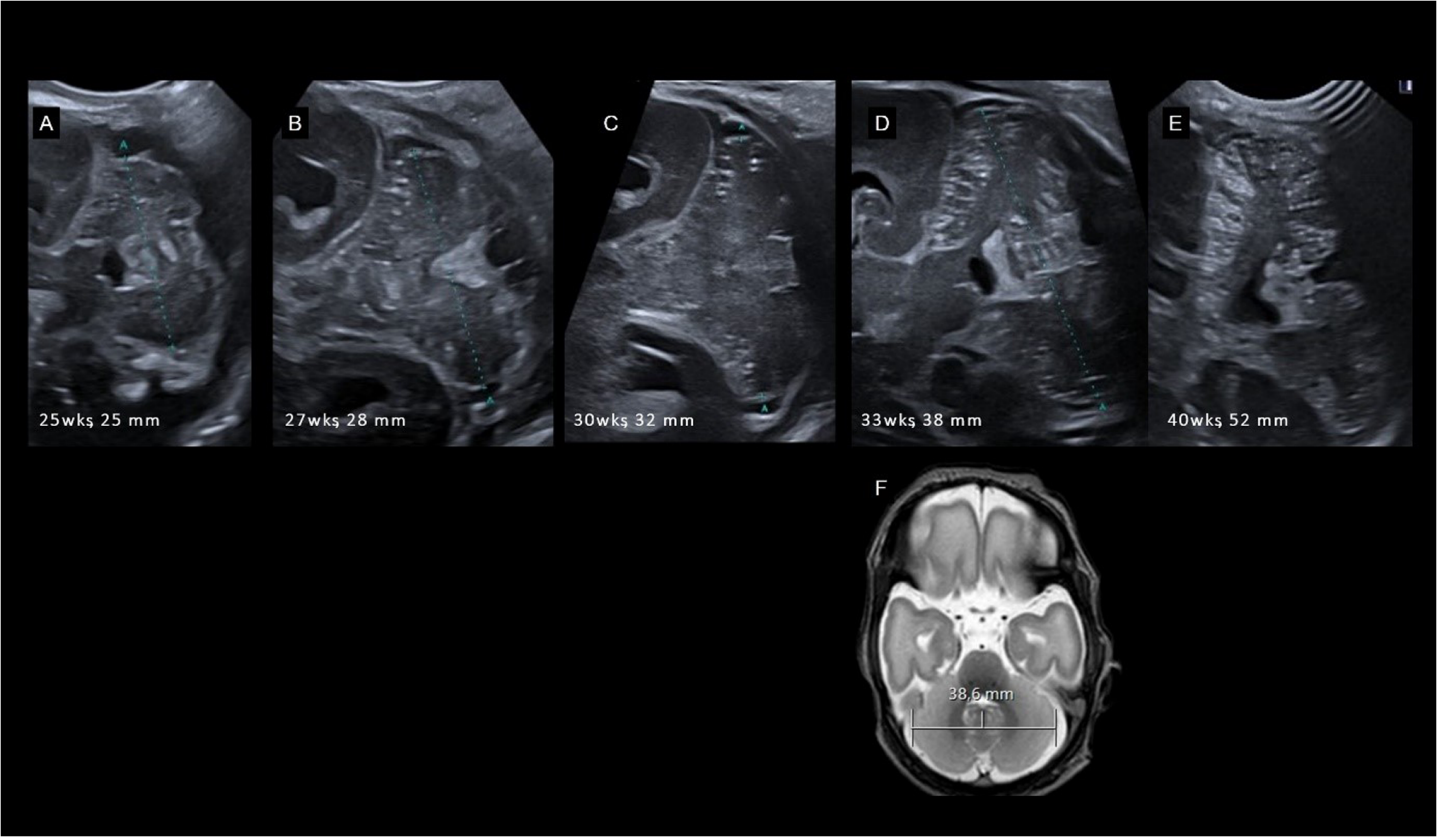
Unimpaired cerebellar growth in a preterm infant of 25 weeks gestational age. Serial coronal ultrasound scans (**A**-**E**) through the mastoid fontanel show a normal development with proceeding foliation representing the rapid increase in cerebellar surface. (**F**) T2 MRI scan at postmenstrual age of 33 weeks results in same transverse cerebellar diameter compared to corresponding ultrasound scan (**D**)

**Fig. 5:**
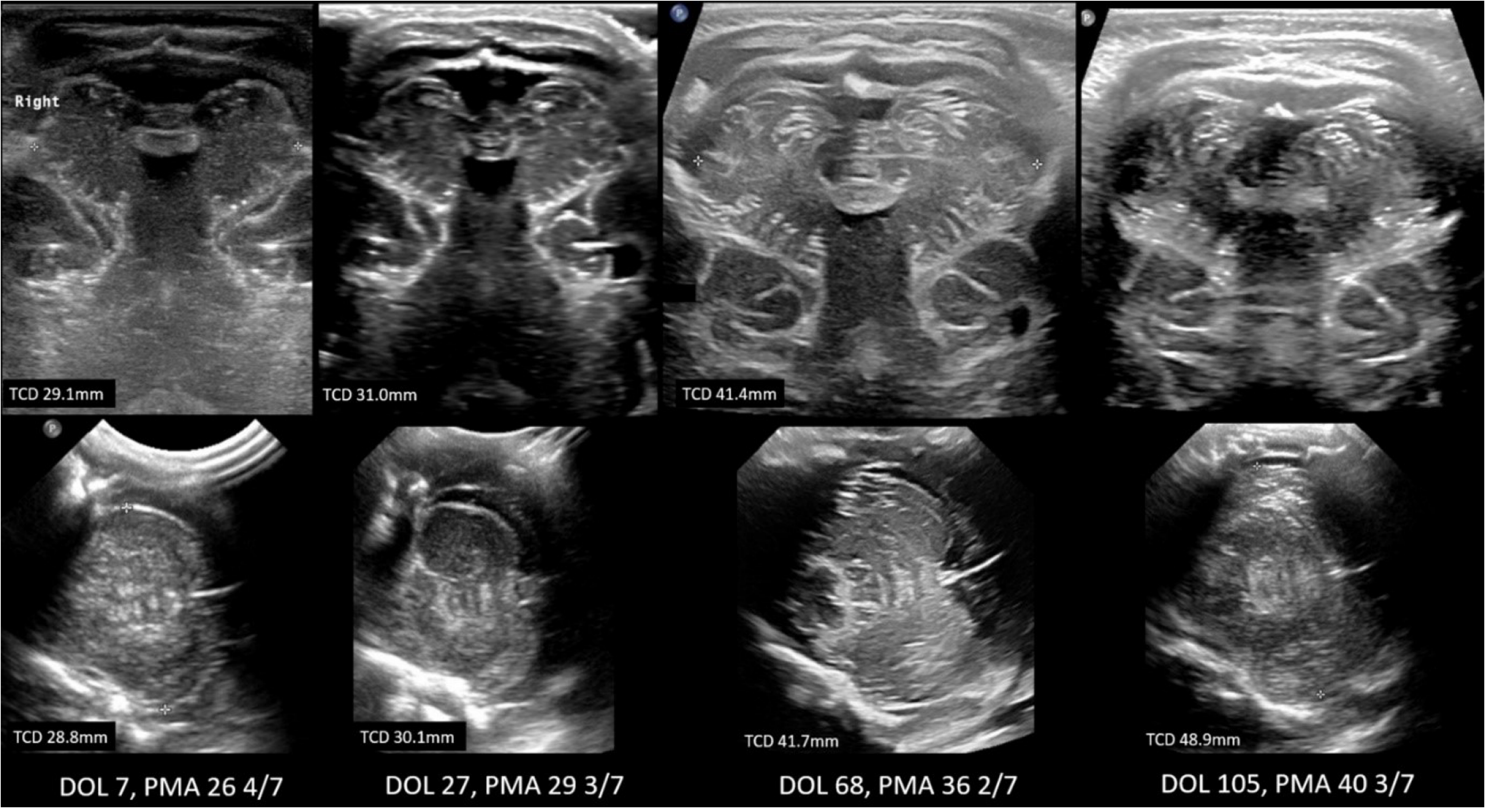
Cerebellar hypoplasia of prematurity: Preterm infant of 25 4/7 weeks gestation age without supra- or infratentorial hemorrhage. Upper row shows serial transnuchal ultrasound scans, the lower row corresponding axial scans through the mastoid fontanel at day of life (DOL) 7 and 27, approximately postmenstrual age (PMA) 36 weeks and term equivalent age (TEA). A normal transverse cerebellar diameter (TCD) of 25-50th percentile according to Imamoglu et al. [29] was present at first scan with one week. Even without recognizable supra- or infratentorial hemorrhage in serial ultrasound scans a mild cerebellar hypoplasia developed during the unremarkable neonatal course with a TCD < 3rd percentile at PMA 36 weeks and TEA

Premature birth, along with associated environmental and intrinsic factors, can disrupt the rapid and intricate development of the cerebellum. Recognizing impaired or altered cerebellar development is important, as it has implications for neurodevelopmental outcome. To detect injury and/or dysmaturation, early neuroimaging of the cerebellum in preterm infants is of particular importance, especially in the most immature infants. Ultrasound is the primary neuroimaging technique to monitor cerebellar growth and maturation because it is safe, can be performed bedside at any time and allows serial scans with minimal stress to the infant [21–23]. To properly examine the cerebellum, additional ultrasound windows should be included in routine scans. Scanning through the mastoid fontanel provides more reliable measurements of the transverse cerebellar diameter (TCD) and better detects cerebellar injury compared to scanning only through the anterior fontanel. This method should be the standard for assessing the cerebellum (Fig. 4) [24–27]. In very low birthweight infants, transnuchal ultrasound via the foramen magnum is feasible and offers the advantage of direct comparison of both cerebellar hemispheres at close range with a high-resolution linear probe; this helps to detect smaller cerebellar lesion and asymmetry (Fig. 5) [23]. Several studies have been published on nomograms for measurements of the TCD and vermis height and diameter [23, 28, 29].

MRI studies offer valuable insights into cerebellar development, connectivity, and the impact of premature birth. However, MRI is resource-intensive, and transporting infants to the scanner can be stressful and is restricted to those in stable condition. As a result, clinical MRI scans are typically performed only at term-equivalent age, with serial scans largely confined to specially designed research studies [10, 30–32]. Nonetheless, MRI has proven superior for the detection of cerebellar lesions compared to ultrasound, especially punctuate cerebellar hemorrhages (CBH), smaller than 4 mm, using susceptibility weighed imaging [27, 33, 34]. Furthermore, MRI offers several other advantages as volumetric measurements, parcellation of the different lobules, examination of cerebellar nuclei, and diffusion tensor imaging (DTI) to assess cerebellar white matter integrity and tractography [10, 30, 31, 35, 36]. Therefore, MRI and ultrasound should be seen as complementary neuroimaging modalities.

### Pathophysiological Mechanisms of Disrupted Cerebellar Development

#### Direct injury: Cerebellar Hemorrhage (CBH) (Figs. 6 and 7)

**Fig. 6:**
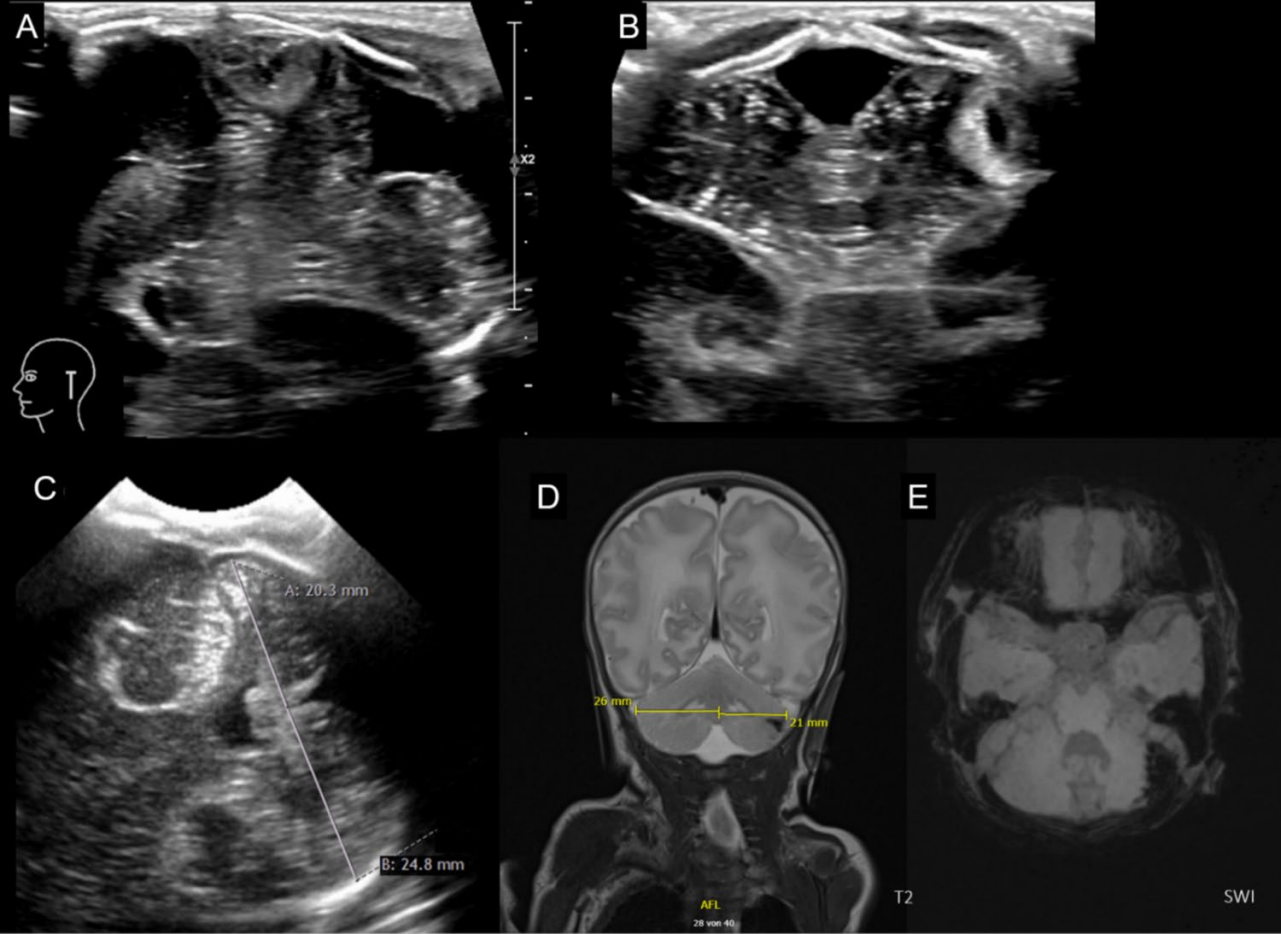
Limited cerebellar hemorrhage (CBH) left posterior-lateral hemisphere in a preterm infant of 29 weeks gestational age. **A** Coronal ultrasound scan through the mastoid fontanel, **B** corresponding transnuchal ultrasound scan on the fourth day of life shows the limited CBH (< 1/3 of the left hemisphere). **C** On the follow-up scan at postmenstrual age (PMA) of 34 5/7 weeks through the left mastoid fontanel, an asymmetrical appearance with smaller left hemisphere can be identified. **D** T2 weighted MRI scan at PMA 36 6/7 weeks and susceptibility weighted imaging (SWI) confirm the asymmetry with a hypoplastic left hemisphere and residual hemosiderin deposition after the CBH

**Fig. 7:**
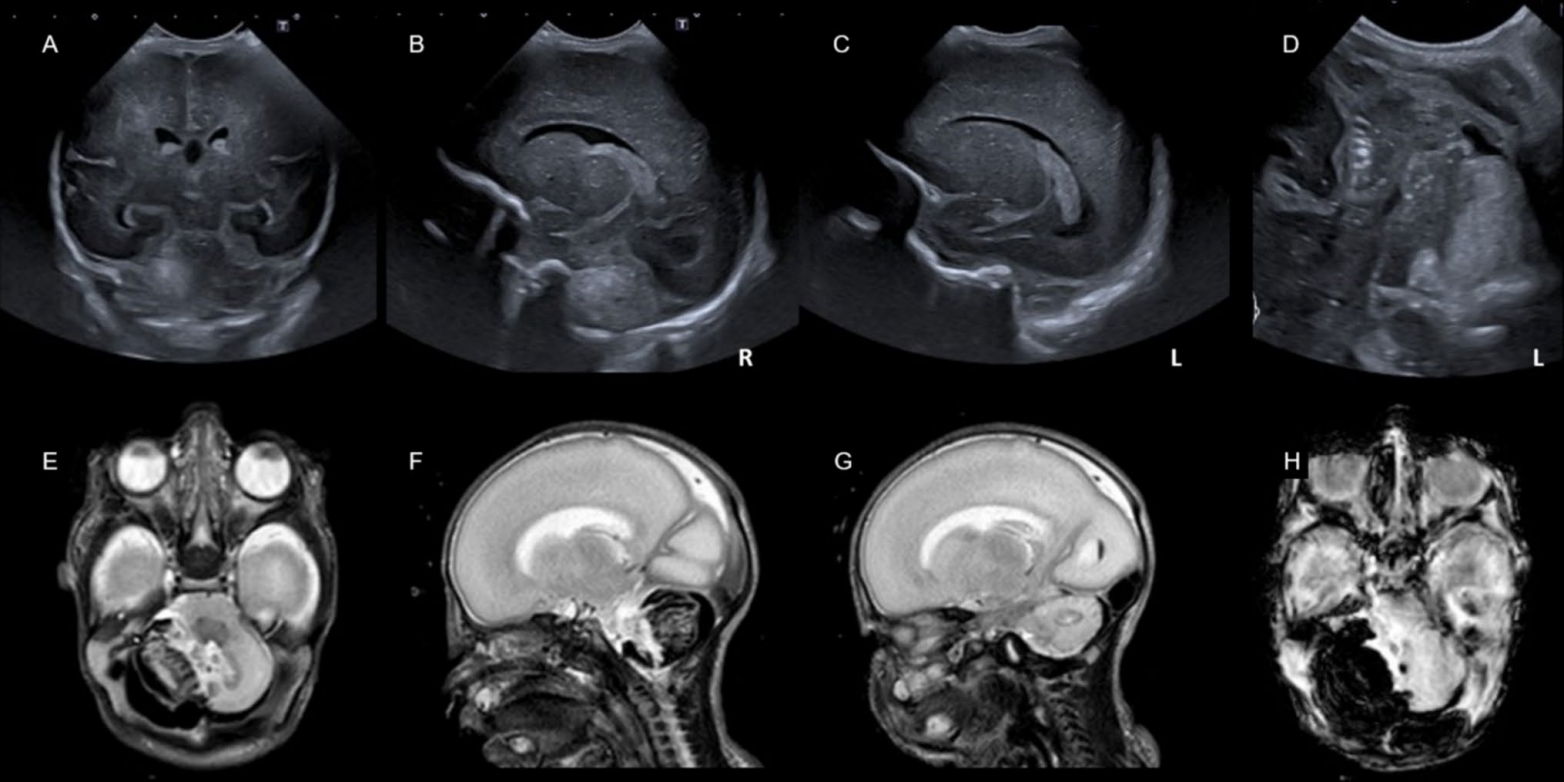
Massive cerebellar hemorrhage (CBH) of the right hemisphere in a preterm infant of 27 weeks gestational age. **A** Coronal scan via the anterior fontanel shows an increased echogenicity (CBH, arrow) in the right cerebellar hemisphere, which can be also seen in (**B**) the right paramedian sagittal scan (arrow). **C** the left cerebellar hemisphere appears normal (arrow). **D** Better visualization of the extend of the massive CBH (> 1/3 of the right hemisphere, arrowheads) via ultrasound scan through the mastoid fontanel with midline shift of the vermis and IV^th^ ventricle. **E** Axial T2 MRI scan and paramedian sagittal T2 scans, **F** corresponding to (**B**) and (**G**) to (**C**), and susceptibility weighted imaging (**H**) proving the massive CBH of the right hemisphere

Infants at highest risk for CBH are often born extremely preterm (< 26–28 weeks) and are in overall worse general condition, e.g., high-frequency ventilation and inotropic support [37–39], therefore CBH is often accompanied by supratentorial hemorrhage [40, 41]. CBH occurs with an incidence of 10 to 38 percent [31, 42, 43]. Small punctate lesions represent around two thirds of CBHs while larger lesions are less common [44]. CBHs are localized most often in the inferior aspect of the posterior lobe as indicated by two MRI studies and a neuropathologic study [31, 41, 43]. The latter study of Haines et al. revealed that the origin of CBH in contrast to supratentorial hemorrhages is usually not the germinal matrix in the ventricular zone or external granular layer (EGL) but the paucicellular internal granular layer (IGL) where rapid vessel growth might cause an increased susceptibility for fluctuations in blood flow or blood pressure leading to rupture of these vessels [41]. Furthermore, histology showed that CBHs are often surrounded by smaller satellite hemorrhages and with evidence for acute and subacute changes which may indicate not only multilocal but also multitemporal onset of CBH [41]. However, as the results of this study are from postmortem investigations, this has to be interpreted with caution as postmortem changes on histologic level might have occurred.

Cerebellar growth and neurodevelopmental outcome are both negatively associated with the size of cerebellar lesions [31, 43–45] and therefore it is reasonable to use a classification system with respect to the size of the primary lesion [44]. The classification used by Boswinkel et al. described massive CBH as hemorrhage affecting more than 1/3 of the respective hemisphere; limited CBH affect less than 1/3 of the hemisphere but are still larger than 4 mm [44]. These massive or limited CBH are usually well detectable on cranial ultrasound via the mastoid fontanel [27, 33]. The punctate cerebellar lesions, less than 4 mm in size, usually require MRI e.g., with susceptibility weighed imaging (SWI) sequences, to be recognized [27, 33]. Neurodevelopmental outcome of patients with punctuate lesions was reported not to be different to infants without CBH in two studies [46, 47], whereas one study found increased odds for worse motor outcome [42] and another study impaired motor function, visuomotor integration and full scale IQ [43]. The CHOPin study compared infants with different sizes of CBH; infants with massive CBH had the worst outcomes even in absence of supratentorial injury [44]. Although not significant, rates of abnormal outcome were higher in infants with bilateral limited or massive CBH or if the vermis was involved. However, infants with limited CBH and punctate CBH had similar outcomes in this study [44]. Apart from the size of the hemorrhage, a separate notion should be made on the number of lesions, laterality, and whether the vermis is affected or not [44, 45, 48]. A sophisticated analysis of size and localization of cerebellar hemorrhages and associated outcomes has been performed by Garfinkle et al. [43]. Of 234 preterm infants born 24 to 32 weeks 36 (15.4%) had CBH, most of them in the inferior posterior lobe. Intriguingly no association of CBH and cognitive function could be shown in this study, but CBH size was independently associated with adverse motor outcomes, visuomotor integration and behavioral outcomes, e.g. externalizing and internalizing behavior. The authors calculated a voxel-wise probabilistic map of CBH and the associated outcomes showing that lesions extending more superiorly and affecting deeper aspects of the cerebellum were more likely to cause adverse outcomes compared to those affecting more superficial parts of inferior regions of the cerebellum [43].

A retrospective study assessed the impact of CBH on neurodevelopmental outcomes in preterm infants < 32 weeks with isolated CBH (identified on ultrasound, confirmed by MRI, *n* = 35) compared to age-matched controls (*n* = 35) and infants with CBH plus supratentorial parenchymal injury (*n* = 16) [48]. Infants with isolated CBH had lower scores for gross- and fine motor function, visual reception, language development, early learning composite as well as adverse behavioral outcomes and higher rate of positive screening for autism spectrum disorders [48]. Additional supratentorial parenchymal injury resulted in worse gross motor function but similar results in all the other evaluated domains. Bilateral CBH and CBH with vermis involvement were significantly associated with more profound disability [48]. Hortensius et al. performed a systematic review of eight studies on infants with isolated cerebellar injury (including the previous mentioned study) to evaluate the effect of isolated CBH on cognitive, motor, language and behavioral outcome [45]. Neurodevelopmental outcome of infants with punctate CBH was comparable to infants without brain injury. However, infants with larger CBH ≥ 4 mm had significantly worse cognitive outcomes (41% vs. 13% for punctate CBH) and motor outcomes (43% vs. 7%) and a severe combined impairment in 46–82%. Vermis involvement resulted in an even worse rate of severe combined impairment (87–93%) [45]. Furthermore, CBH including the vermis had a major impact on behavioral outcomes with severe behavioral impairment in 87% [45].

DTI studies showed the effect of CBH on several white matter tracts reflecting disrupted microstructure in the centrum semiovale, corpus callosum, posterior limb of the internal capsule and the superior and middle cerebellar peduncles [49]. These changes are due to remote transsynaptic effects of CBH on the cerebellar circuitry, leading to the so called crossed cerebello-cerebral diaschisis with to smaller contralateral regional cerebral cortex volumes [50] and retrograde degeneration of the pons [51]. These remote effects will be discussed in detail below.

A study using proton magnetic spectroscopy analyzed metabolite concentrations in the cerebellum on *n* = 53 very preterm infants [52]. Those with moderate-to-severe cerebellar injury had lower concentrations of N-acetylaspartate (NAA), choline (Cho) and creatine (Cr) compared to those with no or only mild cerebellar injury likely reflecting the neuronal loss [52]. Further studies on the metabolic profile may provide more insight, whether this advanced neuroimaging technique could be used as prognostic tool for the long-term outcome.

The presented studies clearly demonstrate the importance of direct cerebellar injury for further development, the necessity to scan the cerebellum and to provide long-term follow-up for those with cerebellar lesions.

#### Indirect Injury: Cerebellar Underdevelopment

Indirect injury due to prematurity itself and its consequences can cause a disrupted cerebellar development or “cerebellar hypoplasia of prematurity” [8] (Fig. 5). The following sections summarize the current literature on the respective influencing factors.

#### Prematurity

Immunohistochemical analysis in an animal model with moderate preterm (equal to 32 weeks in humans) compared to term pigs showed a reduction in granule cell precursors (GCP) and Bergmann glia fibers just by exposure to extrauterine environment [53]. The resulting reduced proliferation but normal differentiation of GCP caused a reduction of mature granule cells (GC) in the IGL [53]. A histopathologic study compared cerebella of preterm infants who died in the neonatal period and still born infants as age-matched “controls” [54]. Comparable to the animal study, deceased preterm infants had a reduced thickness of the EGL with a lower proliferation rate of GCP and decreased sonic-hedgehog (SHH) expression and also decreased Bergmann glia fibers. Maturation of Purkinje cells (PC) or thickness of the molecular layer were not affected by extrauterine environment, but the EGL and IGL showed an increased packing density of the cells [54]. These studies provide immunohistochemical evidence, that the complex programming of cerebellar development might be altered by exposure to the extrauterine environment. However, difficulties in translation of animal models to human (patho-)physiology must be considered.

A resting state functional MRI study of Herzmann et al. showed that corticocerebellar connectivity is already well established at term equivalent age, as well as the circuitry to subcortical or intrinsic networks [55]. Interestingly, infants show more functional connectivity structure within the cerebellum compared to adults, but to a lesser magnitude in preterm infants than in term-born infants [55].

A recent study by Basu et al. compared GABA-edited spectroscopy (MEscher-GArwood Point Resolved Spectroscopy, MEGA-PRESS) in 75 preterm infants without moderate-to-severe brain injury vs. 48 controls to analyze neurometabolic profile in cerebellum, basal ganglia and frontal lobe [56]. Lower concentrations of GABA + , glutamate and NAA in all three regions in moderate preterm infants might indicate a delayed or altered brain maturation due to stressors in the extrauterine environment [56]. However, as GABA + and glutamate concentrations in extremely preterm infants were not different from term infants, the underlying mechanism remains unclear and warrants further studies [56].

#### Hemosiderin or Blood Products (Fig. 8)

The outer part of the EGL, where proliferation of the GCP takes place, is directly exposed to the cerebrospinal fluid of the cisterna magna. Supratentorial intraventricular hemorrhage (IVH) releases red blood cells into the cerebrospinal fluid. Hemoglobin and its metabolites are known to be neurotoxic due to formation of reactive oxygen species and free radicals [57]. Agyemang et al. showed in an animal model that induced IVH leads to an extensive deposition of blood products on the cerebellar surface and even cell-free hemoglobin in the molecular layer and cerebellar white matter. This can lead to an arrest in GCP proliferation with consecutively a smaller EGL and reduced maturation of PC [58]. The detrimental effects on cerebellar development were mitigated by simultaneous injection of haptoglobin as scavenger protein for hemoglobin, demonstrating that hemoglobin is a triggering factor [58]. In another rodent model, intracisternal injection of blood lead to a similar effect on GCP proliferation, EGL thickness and PC density [59]. Additionally, a persisting increase in erratic Bergman glia fiber crossings and decreased molecular layer interneuron density could be demonstrated reflecting the disrupted developmental programming of the cerebellum [59].

**Fig. 8:**
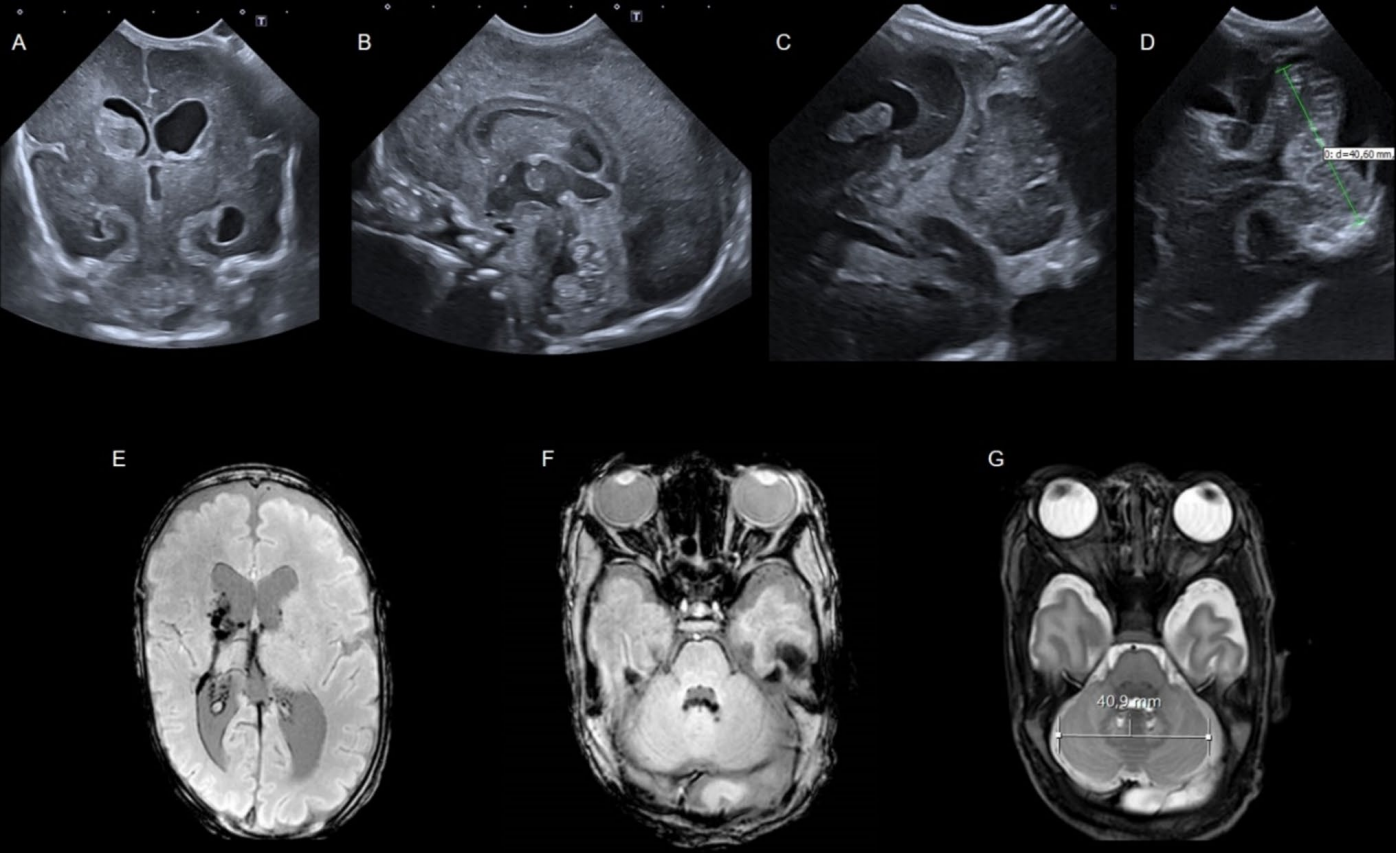
Impaired cerebellar development after high-grade intraventricular hemorrhage in a preterm infant of 25 weeks gestational age. **A** Coronal scan shows IVH III° right side with posthemorrhagic ventricular dilatation. **B** Sagittal midline scan shows an enlarged III^rd^ ventricle and prominent IV^th^ ventricle and blood clots within the posterior fossa around the cerebellar vermis and in the cisterna magna. **C** coronal scan through the mastoid fontanel shows even more pronounced the infratentorial blood clots in the cerebrospinal fluid around the otherwise unaffected cerebellum. **D** coronal scan through the mastoid fontanel at term equivalent age with severe cerebellar hypoplasia, TCD 40.6 mm. (E + F) MRI SWI proves hemosiderin deposition mainly in right lateral ventricle and IV^th^ ventricle, especially the lateral apertures. No parenchymal hemorrhagic infarction in the cerebrum or cerebellum could be identified. **G** T2w scan confirms the symmetric cerebellar hypoplasia seen in US scan

Deposition of hemosiderin on the cerebellar surface was associated with cerebellar hypoplasia also in preterm infants in a MRI study [39]. Further studies have shown that cerebellar growth is impeded in preterm infants with different grades of IVH, often resulting in a symmetric cerebellar hypoplasia [11, 39, 60–62]. This effect was still observed in infants with low grade IVH grade I and II, resulting in smaller cerebellar volumes [62, 63] and disrupted cerebellar white matter tracts with lower fractional anisotropy in the superior and higher apparent diffusion coefficient in the middle cerebellar peduncle [63, 64].

A very recent rodent study examined the effect of fibrinogen on cerebellar development [65]. Injection of plasma containing fibrinogen from wild type mice into the cisterna magna resulted in a disruption of normal cerebellar development compared to injection of fibrinogen depleted plasma from knock-out mice with reduction of cerebellar weight and EGL-area. A further in-vitro analysis showed a dose-dependent inhibition of SHH pathways and GCP proliferation by fibrinogen even at concentration of 0.3 mg/ml, which is lower than even in plasma of extremely preterm infants. Therefore, after higher-grade IVH, not only hemosiderin deposition on the cerebellar surface but also exposition of the EGL to fibrinogen seems to have a negative effect on the cerebellar development [65].

A possible third mechanism involving a direct disruption of the EGL has been reported in a case report of an infant of 25 weeks with severe IVH and fatal course. Neuropathologic and histologic examination showed a disruption of the EGL with the presence of detached GCP cells in the blood around the cerebellum [66]. This mechanism clearly needs more investigation as it has been described only once and as a histopathologic finding, which might be affected by postmortem disruption.

#### Inflammation

Klein et al. demonstrated in a rodent model of systemic inflammation a reduction of total brain volumes and particularly reduced gray and white matter volumes of cerebellar lobules I and II [67]. Immunohistochemical examination revealed an altered myelination and a long-lasting inhibition of oligodendrocyte proliferation and maturation in the cerebellum. This effect is likely driven by cerebellar microglial activation and proliferation responding to the systemic inflammation [67]. Another animal model of CBH and early systemic inflammation also showed a reduction of cerebellar hemisphere and vermis volumes by early inflammation and particularly by combined CBH and early systemic inflammation with altered oligodendrocyte maturation and increased numbers of microglia [68]. A further mechanism of cerebellar hypoplasia has been shown using a mouse model of inflammation with intraperitoneal injection of lipopolysaccharides. Immunohistopathology showed the deposition of fibrinogen in cerebellar parenchyma and surrounding meninges following neurovascular inflammation [65]. During prolonged inflammation, fibrinogen can cross the disrupted blood–brain barrier resulting in inhibition of SHH pathways and GCP proliferation as described above (see CBH) [65].

Another rodent model of cerebral paresis induced by anoxia and sensorimotor restriction caused neuroinflammation with microglial activation in the cerebellum and altered skeletal muscles fibers as well as reduced muscle weight resulting in motor and coordination disorder.[69] Resveratrol, a polyphenol with known neuroprotective and antioxidative properties, could reduce the density of microglia and percentage of microglial activation in the cerebellum and mitigate the abnormal motor function and the skeletal muscle morphology.[69]

Several human studies showed the association of inflammation as sepsis and necrotizing enterocolitis with smaller cerebellar volumes [41, 70, 71]. Kuban et al. measured circulating inflammatory proteins during the first 14 days of life in a prospective cohort of extremely preterm infants and found significantly smaller cerebellar volumes at the age of ten years if at least three inflammatory parameters were elevated [72]. The reduction in cerebellar volume was particularly associated with lower scores in IQ and cognition at ten years.[72] The study of Ranger et al. used constraint principal component analysis of ten clinical predictors, sex and total brain volume to assess the effect of the individual parameters on regional cerebellar volumes. Outcome assessment for the component (weighed set of parameters) with a strong effect of culture proven infections was significantly correlated with Verbal Comprehension and Perceptual Reasoning [71].

#### Pain and Opioid Therapy

The aforementioned study by Ranger et al. identified in addition to the effects of sepsis a negative association between pain, measured by the number of invasive procedures, and the volumes of lobules VIIIA and VIIIB of the cerebellum [71], which are linked to sensorimotor projections [18]. Furthermore, several factors were found to adversely impact these regions, including cumulative exposure to morphine, duration of mechanical ventilation, severity of illness, and exposure to postnatal dexamethasone. Conversely, a higher gestational age at birth was associated with a positive effect on these areas. The study also found that Beery Visual Perception, Verbal Comprehension, and Working Memory scores were correlated with changes in these cerebellar regions [71]. An animal study from the same group examined the effect of exposure to oral sucrose vs. vehicle and pain vs. touch or normal handling on volumes of brain different structures [73]. Volumes of cerebral white matter, corpus callosum, hippocampus and cerebellum were smaller in adult mice treated with sucrose during the first week of life compared to vehicle. Intriguingly, no effect of painful intervention (needle prick) could be shown compared to routine handling. The negative effect of sucrose treatment might be caused by the opioid-like effect on dopamine receptors [73]. A study with the same design modeling pain and sucrose exposure during in the neonatal period showed that adult mice exposed to pain had poorer memory function regardless of treatment with sucrose or vehicle [74]. Sucrose treatment in the absence of painful stimuli caused worse short-term memory function and behavioral changes with lower sugar preference in adult mice [74].

Resting state fMRI at term equivalent age of 148 very preterm infants showed a hyperconnectivity within the cerebellum and between cerebellum and limbic and paralimbic regions compared to 99 term born controls [75]. There was no effect of number of skin breaking procedures as a proxy for pain exposure and regional cerebral or cerebellar volumes. However, intracerebellar connectivity and connectivity of the cerebellum with association cortex, paralimbic and limbic were significantly associated with number of skin breaking procedures. Further analysis of individual ROI to ROI connections showed positive relationships with skin breaking procedures, but no more significant after correction for multiple comparison [75]. At the age of 18 months infants at risk for autism identified by the Modified Checklist of Autism in Toddlers (M-CHAT) had more skin breaking procedures. An interaction of skin breaks and GA at birth with higher M-CHAT-scores suggested that pain exposure in the most preterm infants is associated with an increased risk for autism [75]. A similar complex interaction could be shown for lower expressive language scores and skin breaks and earlier GA, while gross and fine motor function were simply negatively associated with number of skin breaks without effect of the GA [75].

A dose depending negative effect of cumulative morphine dose on cerebellar growth could be shown in very preterm infants; a ten-fold increase in morphine dose led to 5.5 percent lower cerebellar volumes and predicted worse motor and cognitive outcomes at 18 months of life [76]. A recent MRI study could reproduce the negative effect of early morphine exposure (cumulative dose during the first week) on cerebellar volumes and was additionally associated with a higher abnormality score (Kidokoro score [77]) of the cerebellum and white matter [78]. Similarly, the cumulative fentanyl dose during the first week of life correlated with smaller TCD at term equivalent age in a cohort study of preterm infants < 30 weeks, but this study could not show an effect on neurodevelopmental outcome at 2 years of age [79].

#### Postnatal Corticosteroids

Exposure to postnatal corticosteroids is relatively common, especially in the most preterm infants to treat evolving bronchopulmonary dysplasia or adrenal insufficiency [80, 81]. Animal studies show a reduced proliferation of GCP and accelerated maturation of GC by both hydrocortisone and dexamethasone [82, 83]. This might be caused by a downregulation of the SHH pathway and PC death [84]. Administration of a SHH agonist could prevent volume loss and PC death in a rodent model of chronic hypoxia and prednisolone administration [84].

The PREMILOC trial, using prophylactic early hydrocortisone, showed no difference regarding MRI at term equivalent age in hydrocortisone treated infants [85]. Comparable, hydrocortisone had no significant effect in the study of Ranger et al., but dexamethasone treatment was associated with smaller regional cerebellar volumes (lobule VIIIA and VIIIB) [71]. In contrast, Tam et al. described lower cerebellar volumes at term equivalent age after hydrocortisone treatment (1.88 cm^3^ = 8% reduction) and even more pronounced after dexamethasone treatment (2.31 cm^3^ = 10% reduction) [86].

In a recent study, Han-Menz et al. could not show an effect of postnatal corticosteroids on TCD measurement and vermis height on MRI at term equivalent age [87]. However, it has to be noted that the authors used a calculation for hydrocortisone into an equivalent dexamethasone dose what might have affected the outcome, as the above-mentioned studies show conflicting results regarding hydrocortisone.

A similar negative effect on cerebellar growth by both dexamethasone and hydrocortisone treatment for bronchopulmonary dysplasia could be shown in a retrospective ultrasound study by Warmerdam et al. [88]. Adjusting for illness severity, serial TCD measurements before and after corticosteroid therapy demonstrated that slower cerebellar growth was caused by corticosteroid treatment and not by illness severity itself. This is contrasted by cerebral growth, which was not associated with corticosteroid treatment but illness severity [88].

#### Hypoxia and Hyperoxia

Cerebellar susceptibility to hypoxia and hyperoxia could be demonstrated in several rodent models. A chronic hypoxia of 10% leads to cerebellar hypoplasia by decreased GCP proliferation and downregulation of SHH signaling but no signs of apoptosis (in contrast to additional corticosteroid treatment) [84]. High levels of environmental oxygen, i.e., hyperoxia of 80% for 24 h, also led to long-term cerebellar injury with reduced volumes on MRI thirty and sixty days later [89]. Immunohistochemical examination showed an increased apoptosis, reduced proliferation and maturational delay of cerebellar oligodendrocyte precursors [89]. In another study of this group, hyperoxia caused impaired GCP proliferation and apoptosis of immature GC by oxygen toxicity. Furthermore, maturation of the PC dendritic tree was hampered by hyperoxia with an inhibition of SHH production [90]. Zonouzi et. al showed, using a rodent model of hypoxia-induced diffuse white matter injury, not only a decrease in cerebellar white matter volume by reduced myelin formation but also a sophisticated analysis on molecular and cellular level [91]. Hypoxia disrupted the PC development resulting in a smaller dendritic tree and lesser arborization as well as reduced PC function with lower simple-spike firing rates. There were no signs of PC apoptosis or axonal loss. A disruption of gamma-aminobutyric acid (GABA)ergic signaling by loss of proliferation of interneuron progenitor cells led to a delayed maturation of cerebellar oligodendrocyte precursor cells into mature oligodendrocytes eventually leading to hypomyelination of the cerebellar white matter. The authors of this study also presented possible treatment strategies with tiagabine or vigabatrin, which ameliorated the effects of hypoxia on disrupted oligodendrocyte maturation and myelination [91].

These animal models provide evidence for the reduced capacity of immature neurons and glia cells of the cerebellum to cope with decreased and increased levels of oxygen.

#### Patent Ductus Arteriosus

Comparable to IVH, changes in cerebral blood flow are at least a risk factor for CBH but there is also some evidence for an effect of altered cerebral blood flow, e.g., by large patent ductus arteriosus (PDA), on cerebellar growth. Some studies identified a large PDA [86, 92] and PDA ligation [62] being independent risk factors (besides others) for cerebellar hypoplasia. PDA ligation was also associated with a higher mean diffusivity in the middle cerebellar peduncle and vermis reflecting changes in cerebellar microstructure [35].

#### Nutrition

With the rapid development of the cerebellum during the third trimester its growth requires nutritional factors. One very recent randomized controlled trial found smaller cerebellar volumes in preterm infants < 28 weeks and < 1000 g (*n* = 34) treated with multicomponent lipid emulsions compared to soybean-based lipids but no effect on total brain volumes [93]. However, it has to be mentioned that time to full enteral feeding (mean 40 days and 36 days, respectively) was very long and mean caloric intake relatively low with 93 kcal/kg/d during the first 28 days of life [93]. Early enteral nutrition might affect brain development. Terrin et al. found a positive correlation between enteral protein intake during the first week and TCD and vermis height and width on sonographic assessment at 28 days of life [94]. Intriguingly, the authors also found a negative correlation between parenteral protein intake and cerebellar measurements of which only vermis width remained significant in multivariate analysis [94]. A prospective study in very preterm infants demonstrated that enteral feeding with breast milk compared to formula milk improved cerebellar volumes and white matter maturation in the middle cerebellar peduncle as well as microstructural development in the cerebellar vermis with lower fractional anisotropy [95]. Several ingredients in breast milk as growth factors, cholesterol and long-chain polyunsaturated fatty acid and immunomodulating factors might contribute to the growth and maturation of the cerebellum [95]. The association between early cholesterol levels in very preterm infants and larger cerebellar volumes both at early MRI (mean 31.4 weeks) and at term equivalent age was identified by another recent cohort study. This correlation is possibly attributed to upregulated SHH signaling, which may be influenced by higher cholesterol levels [96]. Paviotti et al. reported a positive correlation between postnatal growth defined as mean daily weight gain, fat mass and fat free mass and higher brain volumes especially of the cerebellum on MRI at term equivalent age in a cohort of 42 very low birth weight infants. Postnatal weight gain remained an independent factor in multiple regression analysis for larger cerebellar volumes besides mechanical ventilation and postconceptional age at MRI [97].

#### Early Functional Activity and Transsynaptic Effects

There is increasing evidence for the importance of early brain activity for development of neuronal survival and formation of brain networks [98]. As the cerebellum is an integral part of multiple large networks, [1] it is reasonable that this early activity also has impact on cerebellar growth. Spontaneous activity transients (SAT) are high voltage burst found during the premature period but abruptly disappearing around term age. Tataranno et al. demonstrated that in 33 preterm infants < 28 weeks higher rates of SAT during the first two days of life were correlated with increased cerebellar and cortical gray matter volume and gyrification at MRI at term equivalent age [99]. Furthermore, the duration of inter-burst intervals between these SAT per minute was negatively associated with cerebellar growth [99]. This relationship between early brain activity and later structural brain development has been further investigated in a study of 106 preterm infants < 28 weeks reproducing the strong correlation between cerebellar growth, early brain activity and the complexity index of the EEG [100].

**Fig. 9:**
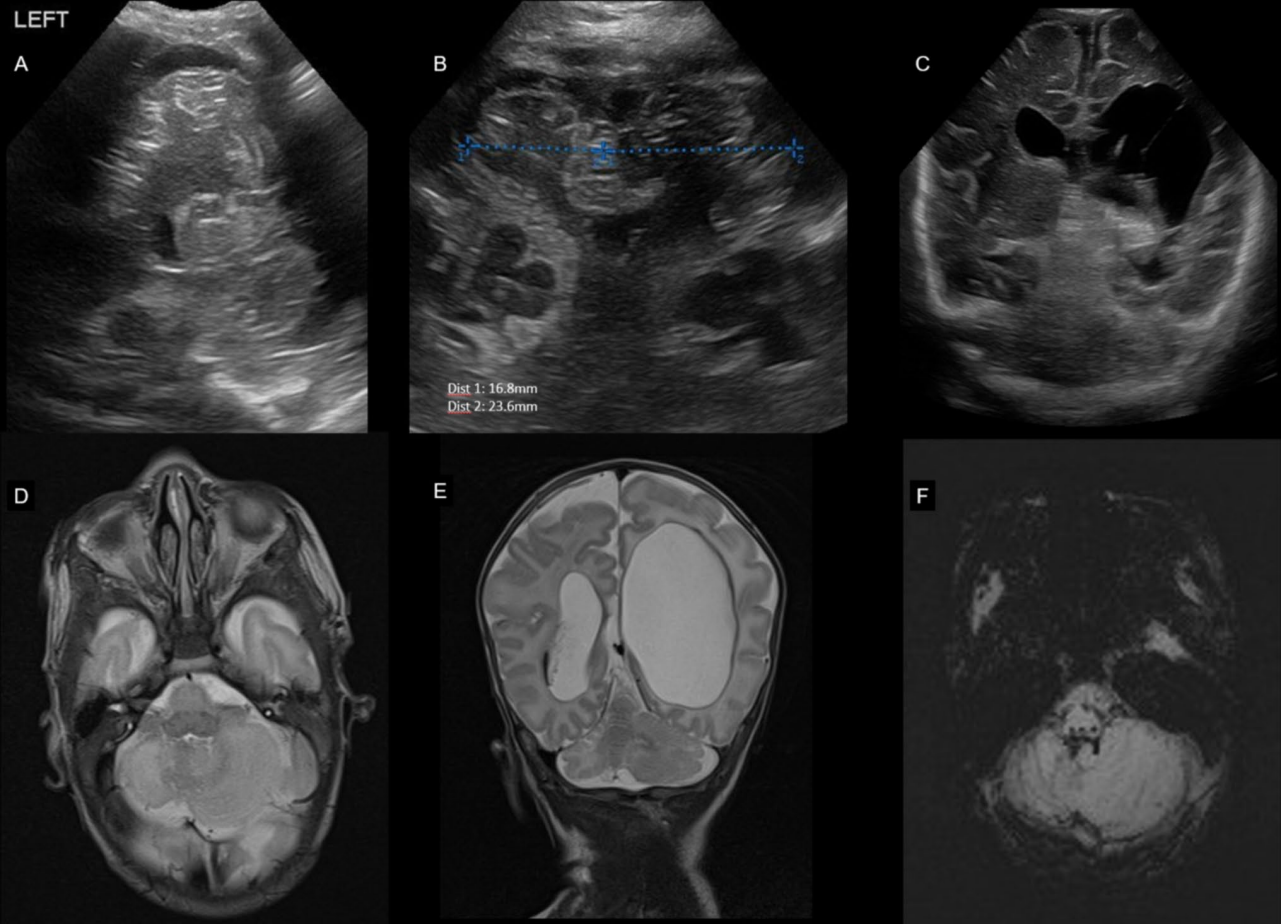
Crossed cerebro-cerebellar diaschisis. Preterm infant 24 weeks gestational age with IVH III and left-sided periventricular hemorrhagic infarction. At postmenstrual age of 38 weeks exists an asymmetry with a hypoplastic contralateral right cerebellar hemisphere (**A**) in ultrasound via mastoid fontanel and (**B**) transnuchal ultrasound scan. **C** Posterior coronal scan shows the extent of the periventricular hemorrhagic infarction with porencephaly. (**D** + **E**) T2 weighted MRI scans are also with hypoplasia of the contralateral cerebellar hemisphere and without signs of a direct cerebellar lesion. (**F**) With no susceptibility artefacts in SWI within the cerebellum or on the cerebellar surface, a crossed cerebro-cerebellar diaschisis is proven (for more information see text)

Disruption of these transsynaptic trophic interactions due to larger supratentorial lesions or cerebellar lesions can cause deleterious effects on the development of the remote areas. Asymmetric cerebellar hypoplasia in absence of direct cerebellar injury with a large supratentorial lesion has already been described in a case series by Rollins et al. [101] Fig. 9 shows ultrasound and MRI features of a preterm infant with crossed cerebro-cerebellar diaschisis. Limperopoulos et al. also reported this phenomenon in a cohort study and, additionally, demonstrated the vice versa effect of cerebellar lesions leading to a reduction of contralateral cerebral volume (crossed cerebello-cerebral diaschisis) [102]. A follow-up study of former preterm infants with isolated cerebellar injury demonstrated on MRI at three years of age an impaired growth of several cerebral regions affecting both gray and white matter [50]. Furthermore, the impeded remote cortical development after isolated cerebellar injury was linked to domain-specific functional deficits in neurodevelopment [103].

Not all studies could reproduce crossed diaschisis between cerebrum and cerebellum. Lower cerebellar volumes have been associated with supratentorial IVH in a study of Tam et al. with stronger effect in more severe grades of IVH, however, the authors reported an effect of IVH on the ipsilateral as well as on the contralateral cerebellar hemisphere [60]. The authors concluded that the symmetry of cerebellar hypoplasia was due to the effect of blood within the cerebrospinal fluid as described above. A more recent study by Dijkshoorn et al. also reported no changes on macroscopic level but the authors could show a cortical thinning and thickening on microlevel in several different cortical areas in both cerebral hemispheres after CBH [104]. The results of this study with the microlevel changes implicate that the cerebello-cerebral interaction and remote development might be even more complex with a widespread bilateral effect on cortical maturation.

### Prenatal and Postnatal Cerebellar Growth

As described before, intrauterine cerebellar growth reaches its peak during the third trimester, from 24 weeks to around term equivalent age [9, 105, 106]. After preterm birth, cerebellar growth is still rapid, but several studies using ultrasound or MRI have reported that postnatal cerebellar growth in very preterm or extremely preterm infants is impeded, resulting in a ‘cerebellar hypoplasia of prematurity’ (Fig. 5) [22, 23, 32, 36, 107].

An MRI study of Brossard-Racine et al. compared intrauterine and extrauterine regional cerebellar growth of preterm infants < 32 weeks < 1500 g (*n* = 38) and full-term controls (*n* = 38) [30]. MRIs in preterm infants were performed as soon as clinically stable. Age-matched (later term-born) controls received intrauterine MRI and both groups underwent a second MRI around term. At the first MRI with mean postmenstrual age of 34 weeks, preterm infants had smaller cerebellar hemispheres and total cerebellar volume with no difference in vermis size; only after correction for total brain volume, hemispheres and vermis volumes both were significantly smaller than in intrauterine controls [30]. At term equivalent age all volumes were smaller in preterm infants, however after correction for total brain volume, only vermis volumes remained significantly smaller. An interesting fact is that the reduced cerebellar volumes occurred already at the first MRI, indicating that the duration of mean 46 days in extrauterine environment had already hampered normal development of the cerebellum [30].

Another recent study of Wu et al. with similar design also found smaller cerebellar hemispheres but larger regional vermis and paravermis volumes and smaller brainstem volumes in very preterm infants at term indicating an altered regional cerebellar and brainstem growth in extrauterine environment [36]. In this study, changes in vermis volumes were evident only at term equivalent age but not at the first MRI at mean 34 weeks postmenstrual age. However, both studies showed an altered regional cerebellar development after preterm birth even without direct cerebellar injury [30, 36].

Regional development of the cerebellum was also altered in an MRI study of 52 preterm infants (median GA 32.4 ± 3.5) compared to 312 term born controls. The shape analysis resulted in an increased thickness of the vermis and lower thickness of the lateral portions of both cerebellar hemispheres in preterm infants compared to term controls, without difference of either total cerebellar volumes, hemispheric volumes or total brain volume [108]. A possible explanation for the lack of significant changes in the volumes might be the small number of extremely preterm (*n* = 5 < 28 weeks) and very preterm (*n* = 18 < 32 weeks) infants in this study. Structural covariance analysis of the cerebellum and frontal and parietal lobe showed a stronger association in preterm compared to term born infants possibly suggesting a similar effect of prematurity on cerebellar and cerebral development, however, the underlying mechanism remains unclear [108].

Further longitudinal follow-up of former preterm infants showed a long-lasting detrimental effect of prematurity on cerebellar growth up to early childhood [11] and even school-age [12, 109–111]. One longitudinal study of Parker et al. found even a significant reduction of 3.11% of the cerebellar volumes in former very preterm infants when scanned at 18–19 years compared to a first MRI at 15 years, but no change in term controls [112]. Another prospective MRI study indicated that the socioeconomical status, measured by highest parental education, might positively influence cerebellar growth during early childhood with maternal, but not paternal postsecondary education, being a significant factor for cerebellar growth between MRI at term and at 2 years of age [113].

Several studies showed differences between girls and boys with cerebellar volumes being smaller in girls, already at 2 years and persisting until school age [72, 110, 114].

### Effect of an Altered Cerebellar Development on the Neurodevelopmental Outcome

As described above, direct cerebellar lesions as CBH are clearly associated with worse combined neurodevelopmental outcomes. However, cerebellar hypoplasia without direct parenchymal injury is also associated with an adverse neurodevelopmental outcome. One study of Spittle et al. demonstrated the association between abnormal general movements at three months of age (which predict later motor outcomes) and smaller cerebellar volumes at term age [115]. In accordance with this finding, several studies report lower/poorer motor performance or coordination disorders at school age [110, 111, 116, 117].

Brossard-Racine et al. used the term developmental cerebellar cognitive-affective syndrome for infants with direct cerebellar injury as CBH or cerebellar infarction [7], but the multimodal impairment in neurodevelopmental outcome has also been shown in studies of premature infants without direct cerebellar lesions. In very preterm infants without brain injury smaller TCD were associated with a lower mental developmental index at two years of age [114]. Matthews et al. compared in a longitudinal study preterm infants < 30 weeks with term controls, using serial MRI at term and at age of seven years, showing both smaller cerebellar volumes and poorer postnatal cerebellar growth in the preterm cohort [110]. Several neurodevelopmental outcomes at seven years including IQ, receptive language and motor function were positively associated with cerebellar volumes at term equivalent age and at seven years. Furthermore, increased cerebellar growth was correlated with better neurodevelopmental outcome at seven years regarding IQ, language, balance and manual dexterity [110]. One study using serial ultrasound scans with linear measurements of cerebral structures and TCD in a prospective cohort of infants < 30 weeks without major brain injury could show the association of larger cerebellar size at two months postnatal age and faster cerebellar growth between one and two months with higher cognitive and language scores at two years of age [118]. A small study compared a cohort consisting of 22 preterm infants born between 28 and 33 weeks and without major morbidities (considered as “low-risk” for neurodevelopmental impairment) with 24 term controls.[109] The “low-risk” cohort still had smaller cerebellar and hippocampal volumes and a smaller corpus callosum area on MRI at nine years of age which correlated with worse attention and executive functions in the preterm group [109].

Not only total cerebellar volumes but also alterations in cerebellar white matter tracts were associated with several impaired outcomes. An MRI DTI study of preterm infants and term controls demonstrated that white matter microstructure of the cerebellar peduncles correlates with visual motor, visual memory and fine motor skills with substantial deficits in the preterm cohort [119].

Stipdonk et al. performed a systematic review to assess language skills in relation to different brain volumes including the cerebellum. Four studies showed conflicting results regarding the effect of cerebellar volumes on language development with two showing a positive correlation and two others showing none [120]. In a more differentiated study, Stipdonk et al. were able to show that language skills were not associated with total cerebellar volume but with volumes of the right hemispheric Crus I + II [121] providing further evidence for the structural–functional topography.

Autism spectrum disorder has a high prevalence in preterm infants [122] and core autism symptoms are associated with regional volume changes in the cerebellum [123]. Perinatal cerebellar injury is the largest non-hereditary risk for autism with a 36-fold increase while prematurity < 32 weeks still increases the risk 7.3-fold [124]. A prospective cohort study of infants < 33 weeks with MRI at term equivalent age were able to show differences of regional cerebellar volumes in subgroups of M-CHAT screening [125]. Infants with critical-positive scores in M-CHAT (at high risk for autism) had reduced bilateral volumes of cerebellar nuclei compared to negative scoring infants and reduced right cerebellar nuclei volumes compared to non-critical positive scoring infants. However, at the age of 4–7 years no significant associations between cerebellar volumes and typical autism traits could be found in any of the three subgroups [125].

Persistent cerebellar dysfunction up to adulthood could be shown in a study of Tran et al. even in the absence of direct cerebellar lesions [126]. Classical eyeblink conditioning, for which cerebellar pathways are crucial, resulted in slower learning rates and a less conditioned response in former preterm infants at school-age and adulthood [126]. Similar persisting functional changes in the cerebellum up to adulthood were shown in a functional MRI study [127]. Despite comparable results of motor planning, initiation, and execution of simple motor tasks, adults formerly born preterm had a significantly higher activation of the right cerebellar hemisphere with an increased blood-oxygen-level-dependent signal reflecting excessive activation during the motor task due to persisting functional neuronal differences after preterm birth [127].

## Summary and Conclusion

The cerebellum has a highly complex developmental programming spanning from first month of gestation to at least two years of age while becoming an integral part of brain-wide networks. Premature birth with its exposure to stressors in the extrauterine environment can disrupt normal cerebellar development with long-lasting negative effects on cerebellar growth, connectivity, and function leading to deficits in multiple neurodevelopmental outcome domains. Screening for cerebellar injury and monitoring cerebellar growth should be standard of neonatal intensive care in high-risk infants, using serial cerebellar ultrasound scans via mastoid fontanel and/or transnuchal via the foramen magnum. MRI examination should at least be performed when cerebellar hypoplasia or cerebellar injury are suspected on ultrasound scans. Identifying preterm infants with cerebellar injury and/or hypoplasia might help to provide early childhood support to improve neurodevelopmental outcome.

## Data Availability

No datasets were generated or analysed during the current study.

## References

[1] Sathyanesan A, Zhou J, Scafidi J, Heck DH, Sillitoe RV, Gallo V. Emerging connections between cerebellar development, behaviour and complex brain disorders. Nat Rev Neurosci. 2019;20(5):298–313. 10.1038/s41583-019-0152-2. 30923348 10.1038/s41583-019-0152-2 PMC7236620

[2] Buckner RL, Krienen FM, Castellanos A, Diaz JC, Yeo BT. The organization of the human cerebellum estimated by intrinsic functional connectivity. J Neurophysiol. 2011;106(5):2322–45. 10.1152/jn.00339.2011. 21795627 10.1152/jn.00339.2011 PMC3214121

[3] Stoodley CJ, Schmahmann JD. Functional topography of the human cerebellum. Handb Clin Neurol. 2018;154:59–70. 10.1016/B978-0-444-63956-1.00004-7. 29903452 10.1016/B978-0-444-63956-1.00004-7

[4] Schmahmann JD. The cerebellum and cognition. Neurosci Lett. 2019;688:62–75. 10.1016/j.neulet.2018.07.005. 29997061 10.1016/j.neulet.2018.07.005

[5] Glickstein M, Strata P, Voogd J. Cerebellum: history. Neuroscience. 2009;162(3):549–59. 10.1016/j.neuroscience.2009.02.054. 19272426 10.1016/j.neuroscience.2009.02.054

[6] Schmahmann JD, Sherman JC. The cerebellar cognitive affective syndrome. Brain. 1998;121(Pt 4):561–79. 10.1093/brain/121.4.561. 9577385 10.1093/brain/121.4.561

[7] Brossard-Racine M, du Plessis AJ, Limperopoulos C. Developmental cerebellar cognitive affective syndrome in ex-preterm survivors following cerebellar injury. Cerebellum. 2015;14(2):151–64. 10.1007/s12311-014-0597-9. 25241880 10.1007/s12311-014-0597-9 PMC4573589

[8] Gano D, Barkovich AJ. Cerebellar hypoplasia of prematurity: Causes and consequences. Handb Clin Neurol. 2019;162:201–16. 10.1016/B978-0-444-64029-1.00009-6. 31324311 10.1016/B978-0-444-64029-1.00009-6

[9] Volpe JJ. Cerebellum of the premature infant: rapidly developing, vulnerable, clinically important. J Child Neurol. 2009;24(9):1085–104. 10.1177/0883073809338067. 19745085 10.1177/0883073809338067 PMC2799249

[10] Kersbergen KJ, Makropoulos A, Aljabar P, et al. Longitudinal Regional Brain Development and Clinical Risk Factors in Extremely Preterm Infants. J Pediatr. Nov2016;178(93–100): e6. 10.1016/j.jpeds.2016.08.024. 10.1016/j.jpeds.2016.08.024 27634629

[11] Lee W, Al-Dossary H, Raybaud C, et al. Longitudinal cerebellar growth following very preterm birth. J Magn Reson Imaging. Jun2016;43(6):1462–73. 10.1002/jmri.25098. 26595366 10.1002/jmri.25098

[12] Pieterman K, White TJ, van den Bosch GE, et al. Cerebellar Growth Impairment Characterizes School-Aged Children Born Preterm without Perinatal Brain Lesions. AJNR Am J Neuroradiol. May2018;39(5):956–62. 10.3174/ajnr.A5589. 29567656 10.3174/ajnr.A5589 PMC7410678

[13] Andersen BB, Korbo L, Pakkenberg B. A quantitative study of the human cerebellum with unbiased stereological techniques. J Comp Neurol. 1992;326(4):549–60. 10.1002/cne.903260405. 1484123 10.1002/cne.903260405

[14] Herculano-Houzel S. The remarkable, yet not extraordinary, human brain as a scaled-up primate brain and its associated cost. Proc Natl Acad Sci USA. 2012;109(Suppl 1):10661–8. 10.1073/pnas.1201895109. 10.1073/pnas.1201895109 PMC3386878 22723358

[15] Govaert P, Triulzi F, Dudink J. The developing brain by trimester. Handb Clin Neurol. 2020;171:245–89. 10.1016/B978-0-444-64239-4.00014-X. 32736754 10.1016/B978-0-444-64239-4.00014-X

[16] Stoodley CJ, Limperopoulos C. Structure-function relationships in the developing cerebellum: Evidence from early-life cerebellar injury and neurodevelopmental disorders. Semin Fetal Neonatal Med. Oct2016;21(5):356–64. 10.1016/j.siny.2016.04.010. 27184461 10.1016/j.siny.2016.04.010 PMC5282860

[17] Palesi F, De Rinaldis A, Castellazzi G, et al. Contralateral cortico-ponto-cerebellar pathways reconstruction in humans in vivo: implications for reciprocal cerebro-cerebellar structural connectivity in motor and non-motor areas. Sci Rep. 2017;7(1):12841. 10.1038/s41598-017-13079-8. 28993670 10.1038/s41598-017-13079-8 PMC5634467

[18] Stoodley CJ, Schmahmann JD. Functional topography in the human cerebellum: a meta-analysis of neuroimaging studies. Neuroimage. 2009;44(2):489–501. 10.1016/j.neuroimage.2008.08.039. 18835452 10.1016/j.neuroimage.2008.08.039

[19] Guell X, Schmahmann J. Cerebellar functional anatomy: a didactic summary based on human FMRI evidence. Cerebellum. 2020;19(1):1–5. 10.1007/s12311-019-01083-9. 31707620 10.1007/s12311-019-01083-9

[20] Voogd J, Glickstein M. The anatomy of the cerebellum. Trends Neurosci. 1998;21(9):370–5. 10.1016/s0166-2236(98)01318-6. 9735944 10.1016/s0166-2236(98)01318-6

[21] Korsten A, Lequin M, Govaert P. Sonographic maturation of third-trimester cerebellar foliation after birth. Pediatr Res. 2006;59(5):695–9. 10.1203/01.pdr.0000214991.07965.0f. 16627884 10.1203/01.pdr.0000214991.07965.0f

[22] Benavente-Fernandez I, Rodriguez-Zafra E, Leon-Martinez J, et al. Normal Cerebellar Growth by Using Three-dimensional US in the Preterm Infant from Birth to Term-corrected Age. Radiology. Jul2018;288(1):254–61. 10.1148/radiol.2018171956. 29613844 10.1148/radiol.2018171956

[23] Muehlbacher T, Schaefer RN, Buss C, Buhrer C, Schmitz T. A closer look at a small brain: transnuchal ultrasound facilitates high-resolution imaging of the cerebellum in preterm infants. Ultraschall Med. 2020. 10.1055/a-1072-5207. Ein genauerer Blick auf ein kleines Gehirn: Transnuchaler Ultraschall fur die hochauflosende Bildgebung des Zerebellums von Fruhgeborenen. 10.1055/a-1072-5207 31914460

[24] da Graca AL, Cardoso KR, da Costa JM, Cowan FM. Assessment of gestational age using cerebellar measurements at cranial ultrasound: what is the best approach? Early Hum Dev. Jan2013;89(1):1–5. 10.1016/j.earlhumdev.2012.07.008. 22835598 10.1016/j.earlhumdev.2012.07.008

[25] Bravo MC, Valverde E. Reliability in cerebellar size assessment by 2D cranial ultrasonography in neonates. Eur J Paediatr Neurol. Jul2017;21(4):610–3. 10.1016/j.ejpn.2017.03.010. 28433244 10.1016/j.ejpn.2017.03.010

[26] Steggerda SJ, van Wezel-Meijler G. Cranial ultrasonography of the immature cerebellum: Role and limitations. Semin Fetal Neonatal Med. Oct2016;21(5):295–304. 10.1016/j.siny.2016.04.011. 27189326 10.1016/j.siny.2016.04.011

[27] Steggerda SJ, Leijser LM, Wiggers-de Bruine FT, van der Grond J, Walther FJ, van Wezel-Meijler G. Cerebellar injury in preterm infants: incidence and findings on US and MR images. Radiology. Jul2009;252(1):190–9. 10.1148/radiol.2521081525. 19420320 10.1148/radiol.2521081525

[28] Swaminathan M, Davies M, Davis P, Betheras F. Transverse cerebellar diameter on cranial ultrasound scan in preterm neonates in an Australian population. J Paediatr Child Health. 1999;35(4):346–9. 10457289

[29] Imamoglu EY, Gursoy T, Ovali F, Hayran M, Karatekin G. Nomograms of cerebellar vermis height and transverse cerebellar diameter in appropriate-for-gestational-age neonates. Early Hum Dev. 2013;89(12):919–23. 10.1016/j.earlhumdev.2013.10.001. 24183100 10.1016/j.earlhumdev.2013.10.001

[30] Brossard-Racine M, McCarter R, Murnick J, Tinkleman L, Vezina G, Limperopoulos C. Early extra-uterine exposure alters regional cerebellar growth in infants born preterm. Neuroimage Clin. 2019;21: 101646. 10.1016/j.nicl.2018.101646. 30630759 10.1016/j.nicl.2018.101646 PMC6412008

[31] Kim H, Gano D, Ho ML, et al. Hindbrain regional growth in preterm newborns and its impairment in relation to brain injury. Hum Brain Mapp. 2016;37(2):678–88. 10.1002/hbm.23058. 26589992 10.1002/hbm.23058 PMC5094861

[32] Limperopoulos C, Soul JS, Gauvreau K, et al. Late gestation cerebellar growth is rapid and impeded by premature birth. Pediatrics. 2005;115(3):688–95. 10.1542/peds.2004-1169. 15741373 10.1542/peds.2004-1169

[33] Parodi A, Rossi A, Severino M, et al. Accuracy of ultrasound in assessing cerebellar haemorrhages in very low birthweight babies. Arch Dis Child Fetal Neonatal Ed. 2015;100(4):F289–92. 10.1136/archdischild-2014-307176. 25637005 10.1136/archdischild-2014-307176

[34] Steggerda SJ, de Bruine FT, Smits-Wintjens VE, Verbon P, Walther FJ, van Wezel-Meijler G. Posterior fossa abnormalities in high-risk term infants: comparison of ultrasound and MRI. Eur Radiol. Sep2015;25(9):2575–83. 10.1007/s00330-015-3665-8. 25899415 10.1007/s00330-015-3665-8 PMC4529447

[35] Brossard-Racine M, Poretti A, Murnick J, et al. Cerebellar microstructural organization is altered by complications of premature birth: a case-control study. J Pediatr Mar. 2017;182:28–33e1. 10.1016/j.jpeds.2016.10.034. 10.1016/j.jpeds.2016.10.034 PMC13159174 27843009

[36] Wu Y, Stoodley C, Brossard-Racine M, et al. Altered local cerebellar and brainstem development in preterm infants. Neuroimage. 2020;213: 116702. 10.1016/j.neuroimage.2020.116702. 32147366 10.1016/j.neuroimage.2020.116702 PMC7165064

[37] Limperopoulos C, Benson CB, Bassan H, et al. Cerebellar hemorrhage in the preterm infant: ultrasonographic findings and risk factors. Pediatrics. 2005;116(3):717–24. 10.1542/peds.2005-0556. 16140713 10.1542/peds.2005-0556

[38] Zayek MM, Benjamin JT, Maertens P, Trimm RF, Lal CV, Eyal FG. Cerebellar hemorrhage: a major morbidity in extremely preterm infants. J Perinatol. 2012;32(9):699–704. 10.1038/jp.2011.185. 22173133 10.1038/jp.2011.185

[39] Messerschmidt A, Prayer D, Brugger PC, et al. Preterm birth and disruptive cerebellar development: assessment of perinatal risk factors. Eur J Paediatr Neurol. 2008;12(6):455–60. 10.1016/j.ejpn.2007.11.003. 18222715 10.1016/j.ejpn.2007.11.003

[40] Tam EWY. Cerebellar injury in preterm infants. Handb Clin Neurol. 2018;155:49–59. 10.1016/B978-0-444-64189-2.00003-2. 29891076 10.1016/B978-0-444-64189-2.00003-2

[41] Haines KM, Wang W, Pierson CR. Cerebellar hemorrhagic injury in premature infants occurs during a vulnerable developmental period and is associated with wider neuropathology. Acta Neuropathol Commun. 2013;1:69. 10.1186/2051-5960-1-69. 24252570 10.1186/2051-5960-1-69 PMC3893422

[42] Tam EW, Rosenbluth G, Rogers EE, et al. Cerebellar hemorrhage on magnetic resonance imaging in preterm newborns associated with abnormal neurologic outcome. J Pediatr. Feb2011;158(2):245–50. 10.1016/j.jpeds.2010.07.049. 20833401 10.1016/j.jpeds.2010.07.049 PMC3010295

[43] Garfinkle J, Guo T, Synnes A, et al. Location and Size of Preterm Cerebellar Hemorrhage and Childhood Development. Ann Neurol. Dec2020;88(6):1095–108. 10.1002/ana.25899. 32920831 10.1002/ana.25899

[44] Boswinkel V, Steggerda SJ, Fumagalli M, et al. The CHOPIn Study: a Multicenter Study on Cerebellar Hemorrhage and Outcome in Preterm Infants. Cerebellum. Dec2019;18(6):989–98. 10.1007/s12311-019-01053-1. 31250213 10.1007/s12311-019-01053-1

[45] Hortensius LM, Dijkshoorn ABC, Ecury-Goossen GM, et al. Neurodevelopmental consequences of preterm isolated cerebellar hemorrhage: a systematic review. Pediatrics. 2018;142(5). 10.1542/peds.2018-0609. 10.1542/peds.2018-0609 30341153

[46] Steggerda SJ, De Bruine FT, van den Berg-Huysmans AA, et al. Small cerebellar hemorrhage in preterm infants: perinatal and postnatal factors and outcome. Cerebellum. 2013;12(6):794–801. 10.1007/s12311-013-0487-6. 23653170 10.1007/s12311-013-0487-6

[47] Van Kooij BJ, Benders MJ, Anbeek P, Van Haastert IC, De Vries LS, Groenendaal F. Cerebellar volume and proton magnetic resonance spectroscopy at term, and neurodevelopment at 2 years of age in preterm infants. Dev Med Child Neurol. 2012;54(3):260–6. 10.1111/j.1469-8749.2011.04168.x. 22211363 10.1111/j.1469-8749.2011.04168.x

[48] Limperopoulos C, Bassan H, Gauvreau K, et al. Does cerebellar injury in premature infants contribute to the high prevalence of long-term cognitive, learning, and behavioral disability in survivors? Pediatrics. 2007;120(3):584–93. 10.1542/peds.2007-1041. 17766532 10.1542/peds.2007-1041

[49] Neubauer V, Djurdjevic T, Griesmaier E, Biermayr M, Gizewski ER, Kiechl-Kohlendorfer U. The Cerebellar-Cerebral Microstructure Is Disrupted at Multiple Sites in Very Preterm Infants with Cerebellar Haemorrhage. Neonatology. 2018;113(2):93–9. 10.1159/000480695. 29131075 10.1159/000480695

[50] Limperopoulos C, Chilingaryan G, Guizard N, Robertson RL, Du Plessis AJ. Cerebellar injury in the premature infant is associated with impaired growth of specific cerebral regions. Pediatr Res. Aug2010;68(2):145–50. 10.1203/00006450-201011001-0028210.1203/PDR.0b013e3181e1d032. 20389260 10.1203/PDR.0b013e3181e1d032

[51] Parodi A, Ramenghi LA, Malova M, et al. Crossed Pontine Hemiatrophy Associated with Unilateral Cerebellar Hemorrhage in Premature Infants. Neuropediatrics. Dec2016;47(6):404–7. 10.1055/s-0036-1587595. 27552027 10.1055/s-0036-1587595

[52] Basu SK, Pradhan S, Kapse K, et al. Third trimester cerebellar metabolite concentrations are decreased in very premature infants with structural brain injury. Sci Rep. 2019;9(1):1212. 10.1038/s41598-018-37203-4. 10.1038/s41598-018-37203-4 PMC6362247 30718546

[53] Iskusnykh IY, Buddington RK, Chizhikov VV. Preterm birth disrupts cerebellar development by affecting granule cell proliferation program and Bergmann glia. Exp Neurol. Aug2018;306:209–21. 10.1016/j.expneurol.2018.05.015. 29772246 10.1016/j.expneurol.2018.05.015 PMC6291230

[54] Haldipur P, Bharti U, Alberti C, et al. Preterm delivery disrupts the developmental program of the cerebellum. PLoS ONE. 2011;6(8): e23449. 10.1371/journal.pone.0023449. 21858122 10.1371/journal.pone.0023449 PMC3157376

[55] Herzmann CS, Snyder AZ, Kenley JK, Rogers CE, Shimony JS, Smyser CD. Cerebellar functional connectivity in term- and very preterm-born infants. Cereb Cortex. 2019;29(3):1174–84. 10.1093/cercor/bhy023. 29420701 10.1093/cercor/bhy023 PMC6373668

[56] Basu SK, Pradhan S, Sharker YM, et al. Severity of prematurity and age impact early postnatal development of GABA and glutamate systems. Cereb Cortex. 2023;33(12):7386–94. 10.1093/cercor/bhad046. 36843135 10.1093/cercor/bhad046 PMC10267637

[57] Lee JY, Keep RF, He Y, Sagher O, Hua Y, Xi G. Hemoglobin and iron handling in brain after subarachnoid hemorrhage and the effect of deferoxamine on early brain injury. J Cereb Blood Flow Metab. Nov2010;30(11):1793–803. 10.1038/jcbfm.2010.137. 20736956 10.1038/jcbfm.2010.137 PMC2970675

[58] Agyemang AA, Sveinsdottir K, Vallius S, et al. Cerebellar exposure to cell-free hemoglobin following preterm intraventricular hemorrhage: causal in cerebellar damage? Transl Stroke Res. 2017;8(5):461–73. 10.1007/s12975-017-0539-1. 28601919 10.1007/s12975-017-0539-1 PMC5590031

[59] Butler DF, Skibo J, Traudt CM, Millen KJ. Neonatal subarachnoid hemorrhage disrupts multiple aspects of cerebellar development. Front Mol Neurosci. 2023;16: 1161086. 10.3389/fnmol.2023.1161086. 37187957 10.3389/fnmol.2023.1161086 PMC10175619

[60] Tam EW, Miller SP, Studholme C, et al. Differential effects of intraventricular hemorrhage and white matter injury on preterm cerebellar growth. J Pediatr. 2011;158(3):366–71. 10.1016/j.jpeds.2010.09.005. 20961562 10.1016/j.jpeds.2010.09.005 PMC3025266

[61] Sancak S, Gursoy T, Karatekin G, Ovali F. Effect of Intraventricular hemorrhage on cerebellar growth in preterm neonates. Cerebellum. 2017;16(1):89–94. 10.1007/s12311-016-0766-0. 26924821 10.1007/s12311-016-0766-0

[62] Padilla N, Alexandrou G, Blennow M, Lagercrantz H, Aden U. Brain growth gains and losses in extremely preterm infants at term. Cereb Cortex. 2015;25(7):1897–905. 10.1093/cercor/bht431. 24488941 10.1093/cercor/bht431

[63] Jeong HJ, Shim SY, Cho HJ, Cho SJ, Son DW, Park EA. Cerebellar Development in preterm infants at term-equivalent age is impaired after low-grade intraventricular hemorrhage. J Pediatr. 2016;175(86–92): e2. 10.1016/j.jpeds.2016.05.010. 10.1016/j.jpeds.2016.05.010 27283462

[64] Morita T, Morimoto M, Yamada K, et al. Low-grade intraventricular hemorrhage disrupts cerebellar white matter in preterm infants: evidence from diffusion tensor imaging. Neuroradiology. May2015;57(5):507–14. 10.1007/s00234-015-1487-7. 25596864 10.1007/s00234-015-1487-7

[65] Weaver O, Gano D, Zhou Y, et al. Fibrinogen inhibits sonic hedgehog signaling and impairs neonatal cerebellar development after blood-brain barrier disruption. Proc Natl Acad Sci U S A. Jul 30 2024;121(31):e2323050121. doi: 10.1073/pnas.2323050121. 39042684 10.1073/pnas.2323050121 PMC11295022

[66] Calandrino A, Minghetti D, Brigati G, Parodi A, Nozza P, Ramenghi LA. Disruption of Cerebellar Granular Layer as a Consequence of Germinal Matrix Intraventricular Hemorrhage in Extreme Prematurity: An Acute Direct Mechanism Too? Pediatr Neurol. Mar2024;152:20–2. 10.1016/j.pediatrneurol.2023.12.004. 38176224 10.1016/j.pediatrneurol.2023.12.004

[67] Klein L, Van Steenwinckel J, Fleiss B, et al. A unique cerebellar pattern of microglia activation in a mouse model of encephalopathy of prematurity. Glia. Sep2022;70(9):1699–719. 10.1002/glia.24190. 35579329 10.1002/glia.24190 PMC9545095

[68] Tremblay S, Pai A, Richter L, et al. Systemic inflammation combined with neonatal cerebellar haemorrhage aggravates long-term structural and functional outcomes in a mouse model. Brain Behav Immun. Nov2017;66:257–76. 10.1016/j.bbi.2017.07.013. 28755859 10.1016/j.bbi.2017.07.013

[69] Pereira SDC, Manhaes-de-Castro R, Souza VDS, et al. Neonatal resveratrol treatment in cerebral palsy model recovers neurodevelopment impairments by restoring the skeletal muscle morphology and decreases microglial activation in the cerebellum. Exp Neurol. 2024;378: 114835. 10.1016/j.expneurol.2024.114835. 38789024 10.1016/j.expneurol.2024.114835

[70] Lee I, Neil JJ, Huettner PC, et al. The impact of prenatal and neonatal infection on neurodevelopmental outcomes in very preterm infants. J Perinatol. 2014;34(10):741–7. 10.1038/jp.2014.79. 25033076 10.1038/jp.2014.79 PMC4180799

[71] Ranger M, Zwicker JG, Chau CM, et al. Neonatal pain and infection relate to smaller cerebellum in very preterm children at school age. J Pediatr. 2015;167(2):292-8 e1. 10.1016/j.jpeds.2015.04.055. 25987534 10.1016/j.jpeds.2015.04.055

[72] Kuban KCK, Jara H, O’Shea TM, et al. Association of circulating proinflammatory and anti-inflammatory protein biomarkers in extremely preterm born children with subsequent brain magnetic resonance imaging volumes and cognitive function at age 10 years. J Pediatr. 2019;210:81-90.e3. 10.1016/j.jpeds.2019.03.018. 31076229 10.1016/j.jpeds.2019.03.018 PMC7137312

[73] Tremblay S, Ranger M, Chau CMY, et al. Repeated exposure to sucrose for procedural pain in mouse pups leads to long-term widespread brain alterations. Pain. 2017;158(8):1586–98. 10.1097/j.pain.0000000000000961. 28715355 10.1097/j.pain.0000000000000961 PMC5539923

[74] Ranger M, Tremblay S, Chau CMY, Holsti L, Grunau RE, Goldowitz D. Adverse Behavioral Changes in Adult Mice Following Neonatal Repeated Exposure to Pain and Sucrose. Front Psychol. 2018;9: 2394. 10.3389/fpsyg.2018.02394. 30719013 10.3389/fpsyg.2018.02394 PMC6348336

[75] Cook KM, De Asis-Cruz J, Kim JH, et al. Experience of early-life pain in premature infants is associated with atypical cerebellar development and later neurodevelopmental deficits. BMC Med. 2023;21(1):435. 10.1186/s12916-023-03141-w. 37957651 10.1186/s12916-023-03141-w PMC10644599

[76] Zwicker JG, Miller SP, Grunau RE, et al. Smaller cerebellar growth and poorer neurodevelopmental outcomes in very preterm infants exposed to neonatal morphine. J Pediatr. 2016;172:81–87 e2. 10.1016/j.jpeds.2015.12.024. 10.1016/j.jpeds.2015.12.024 PMC5462546 26763312

[77] Kidokoro H, Neil JJ, Inder TE. New MR imaging assessment tool to define brain abnormalities in very preterm infants at term. AJNR Am J Neuroradiol Nov-Dec. 2013;34(11):2208–14. 10.3174/ajnr.A3521. 23620070 10.3174/ajnr.A3521 PMC4163698

[78] Al-Mouqdad MM, Jamjoom DZ, Huseynova R, et al. Association between morphine exposure and impaired brain development on term-equivalent age brain magnetic resonance imaging in very preterm infants. Sci Rep. 2022;12(1):4498. 10.1038/s41598-022-08677-0 35296792 10.1038/s41598-022-08677-0 PMC8927102

[79] McPherson C, Haslam M, Pineda R, Rogers C, Neil JJ, Inder TE. Brain Injury and Development in Preterm Infants Exposed to Fentanyl. Ann Pharmacother. Dec2015;49(12):1291–7. 10.1177/1060028015606732. 26369570 10.1177/1060028015606732 PMC4644677

[80] Doyle LW, Cheong JL, Hay S, Manley BJ, Halliday HL. Late (>/= 7 days) systemic postnatal corticosteroids for prevention of bronchopulmonary dysplasia in preterm infants. Cochrane Database Syst Rev. 2021;11(11):CD001145. 10.1002/14651858.CD001145.pub5. 10.1002/14651858.CD001145.pub5 PMC8580679 34758507

[81] Baud O, Maury L, Lebail F, et al. Effect of early low-dose hydrocortisone on survival without bronchopulmonary dysplasia in extremely preterm infants (PREMILOC): a double-blind, placebo-controlled, multicentre, randomised trial. Lancet. 2016;387(10030):1827–36. 10.1016/S0140-6736(16)00202-6. 26916176 10.1016/S0140-6736(16)00202-6

[82] Aden P, Goverud I, Liestol K, et al. Low-potency glucocorticoid hydrocortisone has similar neurotoxic effects as high-potency glucocorticoid dexamethasone on neurons in the immature chicken cerebellum. Brain Res. 2008;1236:39–48. 10.1016/j.brainres.2008.07.095. 18706896 10.1016/j.brainres.2008.07.095

[83] Aden P, Paulsen RE, Maehlen J, et al. Glucocorticoids dexamethasone and hydrocortisone inhibit proliferation and accelerate maturation of chicken cerebellar granule neurons. Brain Res. 2011;1418:32–41. 10.1016/j.brainres.2011.08.053. 21925649 10.1016/j.brainres.2011.08.053

[84] Nguyen V, Sabeur K, Maltepe E, Ameri K, Bayraktar O, Rowitch DH. Sonic hedgehog agonist protects against complex neonatal cerebellar injury. Cerebellum. 2018;17(2):213–27. 10.1007/s12311-017-0895-0. 29134361 10.1007/s12311-017-0895-0 PMC5849674

[85] Alison M, Tilea B, Toumazi A, et al. Prophylactic hydrocortisone in extremely preterm infants and brain MRI abnormality. Arch Dis Child Fetal Neonatal Ed. 2020;105(5):520–5. 10.1136/archdischild-2019-317720. 31980445 10.1136/archdischild-2019-317720

[86] Tam EW, Chau V, Ferriero DM, et al. Preterm cerebellar growth impairment after postnatal exposure to glucocorticoids. Sci Transl Med. 2011;3(105):105ra105. 10.1126/scitranslmed.3002884. 22013125 10.1126/scitranslmed.3002884 PMC3682111

[87] Han-Menz C, Whiteley G, Evans R, Razak A, Malhotra A. Systemic postnatal corticosteroids and magnetic resonance imaging measurements of corpus callosum and cerebellum of extremely preterm infants. J Paediatr Child Health. 2023;59(2):282–7. 10.1111/jpc.16286. 36404722 10.1111/jpc.16286 PMC10098787

[88] Warmerdam LA, van Wezel-Meijler G, de Vries LS, Groenendaal F, Steggerda SJ. The association of dexamethasone and hydrocortisone with cerebellar growth in premature infants. Neonatology. 2023;120(5):615–23. 10.1159/000531075. 37379806 10.1159/000531075

[89] Scheuer T, Brockmoller V, Blanco Knowlton M, et al. Oligodendroglial maldevelopment in the cerebellum after postnatal hyperoxia and its prevention by minocycline. Glia. 2015;63(10):1825–39. 10.1002/glia.22847. 25964099 10.1002/glia.22847 PMC4534324

[90] Scheuer T, Sharkovska Y, Tarabykin V, et al. Neonatal Hyperoxia Perturbs Neuronal Development in the Cerebellum. Mol Neurobiol. May2018;55(5):3901–15. 10.1007/s12035-017-0612-5. 28547531 10.1007/s12035-017-0612-5

[91] Zonouzi M, Scafidi J, Li P, et al. GABAergic regulation of cerebellar NG2 cell development is altered in perinatal white matter injury. Nat Neurosci. May2015;18(5):674–82. 10.1038/nn.3990. 25821912 10.1038/nn.3990 PMC4459267

[92] Argyropoulou MI, Xydis V, Drougia A, et al. MRI measurements of the pons and cerebellum in children born preterm; associations with the severity of periventricular leukomalacia and perinatal risk factors. Neuroradiology. Oct2003;45(10):730–4. 10.1007/s00234-003-1067-0. 12942217 10.1007/s00234-003-1067-0

[93] Costa S, Cocca C, D'Apolito G, et al. Effects of a Multicomponent Lipid Emulsion on Brain Volumes in Extremely Low Birth Weight Infants. Am J Perinatol. 2023. https://doi.org/10.1055/a-2077-2551. 10.1055/a-2077-2551 37075786

[94] Terrin G, De Nardo MC, Boscarino G, et al. Early protein intake influences neonatal brain measurements in preterms: an observational study. Front Neurol. 2020;11: 885. 10.3389/fneur.2020.00885. 32982918 10.3389/fneur.2020.00885 PMC7479306

[95] Ottolini KM, Andescavage N, Kapse K, Jacobs M, Limperopoulos C. Improved brain growth and microstructural development in breast milk-fed very low birth weight premature infants. Acta Paediatr. 2020;109(8):1580–7. 10.1111/apa.15168. 31922288 10.1111/apa.15168 PMC7347461

[96] Kamino D, Chau V, Studholme C, et al. Plasma cholesterol levels and brain development in preterm newborns. Pediatr Res. 2019;85(3):299–304. 10.1038/s41390-018-0260-0. 30635642 10.1038/s41390-018-0260-0 PMC6433157

[97] Paviotti G, De Cunto A, Zennaro F, et al. Higher growth, fat and fat-free masses correlate with larger cerebellar volumes in preterm infants at term. Acta Paediatr. J 2017;106(6):918–25. 10.1111/apa.13829. 28295577 10.1111/apa.13829

[98] Benders MJ, Palmu K, Menache C, et al. Early brain activity relates to subsequent brain growth in premature infants. Cereb Cortex. 2015;25(9):3014–24. 10.1093/cercor/bhu097. 24867393 10.1093/cercor/bhu097

[99] Tataranno ML, Claessens NHP, Moeskops P, et al. Changes in brain morphology and microstructure in relation to early brain activity in extremely preterm infants. Pediatr Res. 2018;83(4):834–42. 10.1038/pr.2017.314. 29244803 10.1038/pr.2017.314

[100] De Wel O, Van Huffel S, Lavanga M, et al. Relationship between early functional and structural brain developments and brain injury in preterm infants. Cerebellum. 2021;20(4):556–68. 10.1007/s12311-021-01232-z. 33532923 10.1007/s12311-021-01232-z PMC8360868

[101] Rollins NK, Wen TS, Dominguez R. Crossed cerebellar atrophy in children: a neurologic sequela of extreme prematurity. Pediatr Radiol. 1995;25(Suppl 1):S20–5. 8577528

[102] Limperopoulos C, Soul JS, Haidar H, et al. Impaired trophic interactions between the cerebellum and the cerebrum among preterm infants. Pediatrics. 2005;116(4):844–50. 10.1542/peds.2004-2282. 16199692 10.1542/peds.2004-2282

[103] Limperopoulos C, Chilingaryan G, Sullivan N, Guizard N, Robertson RL, du Plessis AJ. Injury to the premature cerebellum: outcome is related to remote cortical development. Cereb Cortex. 2014;24(3):728–36. 10.1093/cercor/bhs354. 23146968 10.1093/cercor/bhs354 PMC3920767

[104] Dijkshoorn ABC, Turk E, Hortensius LM et al. Preterm infants with isolated cerebellar hemorrhage show bilateral cortical alterations at term equivalent age. Sci Rep.2020;10(1):5283. 10.1038/s41598-020-62078-9 10.1038/s41598-020-62078-9 PMC7093404 32210267

[105] Chavez MR, Ananth CV, Smulian JC, Lashley S, Kontopoulos EV, Vintzileos AM. Fetal transcerebellar diameter nomogram in singleton gestations with special emphasis in the third trimester: a comparison with previously published nomograms. Am J Obstet Gynecol. Oct2003;189(4):1021–5. 10.1067/s0002-9378(03)00894-9. 14586348 10.1067/s0002-9378(03)00894-9

[106] Chavez MR, Ananth CV, Smulian JC, Yeo L, Oyelese Y, Vintzileos AM. Fetal transcerebellar diameter measurement with particular emphasis in the third trimester: a reliable predictor of gestational age. Am J Obstet Gynecol. Sep2004;191(3):979–84. 10.1016/j.ajog.2004.06.046. 15467576 10.1016/j.ajog.2004.06.046

[107] Parikh NA, Lasky RE, Kennedy KA, McDavid G, Tyson JE. Perinatal factors and regional brain volume abnormalities at term in a cohort of extremely low birth weight infants. PLoS ONE. 2013;8(5): e62804. 10.1371/journal.pone.0062804. 23671636 10.1371/journal.pone.0062804 PMC3650008

[108] Xu F, Wang Y, Wang W, Liang W, Tang Y, Liu S. Preterm birth alters the regional development and structural covariance of cerebellum at term-equivalent age. Cerebellum. 2024. 10.1007/s12311-024-01691-0 10.1007/s12311-024-01691-0 38581612

[109] Arhan E, Gucuyener K, Soysal S, et al. Regional brain volume reduction and cognitive outcomes in preterm children at low risk at 9 years of age. Childs Nerv Syst. Aug2017;33(8):1317–26. 10.1007/s00381-017-3421-2. 28484867 10.1007/s00381-017-3421-2

[110] Matthews LG, Inder TE, Pascoe L, et al. Longitudinal Preterm Cerebellar Volume: Perinatal and Neurodevelopmental Outcome Associations. Cerebellum. Oct2018;17(5):610–27. 10.1007/s12311-018-0946-1. 29949094 10.1007/s12311-018-0946-1 PMC6126980

[111] Dewey D, Thompson DK, Kelly CE, et al. Very preterm children at risk for developmental coordination disorder have brain alterations in motor areas. Acta Paediatr. 2019;108(9):1649–60. 10.1111/apa.14786. 30891804 10.1111/apa.14786

[112] Parker J, Mitchell A, Kalpakidou A, et al. Cerebellar growth and behavioural & neuropsychological outcome in preterm adolescents. Brain. 2008;131(Pt 5):1344–51. 10.1093/brain/awn062. 18372312 10.1093/brain/awn062

[113] Stiver ML, Kamino D, Guo T, et al. Maternal postsecondary education associated with improved cerebellar growth after preterm birth. J Child Neurol. 2015;30(12):1633–9. 10.1177/0883073815576790. 25818328 10.1177/0883073815576790

[114] Hammerl M, Zagler M, Griesmaier E, et al. Reduced cerebellar size at term-equivalent age is related to a 17% lower mental developmental index in very preterm infants without brain injury. Neonatology. 2020;117(1):57–64. 10.1159/000502491. 31480070 10.1159/000502491

[115] Spittle AJ, Doyle LW, Anderson PJ, et al. Reduced cerebellar diameter in very preterm infants with abnormal general movements. Early Hum Dev. 2010;86(1):1–5. 10.1016/j.earlhumdev.2009.11.002. 20004536 10.1016/j.earlhumdev.2009.11.002

[116] Anderson PJ, Treyvaud K, Neil JJ, et al. Associations of newborn brain magnetic resonance imaging with long-term neurodevelopmental impairments in very preterm children. J Pediatr. 2017;187:58–65e1. 10.1016/j.jpeds.2017.04.059. 10.1016/j.jpeds.2017.04.059 PMC5533625 28583705

[117] Bolk J, Padilla N, Forsman L, Brostrom L, Hellgren K, Aden U. Visual-motor integration and fine motor skills at 6(1/2) years of age and associations with neonatal brain volumes in children born extremely preterm in Sweden: a population-based cohort study. BMJ Open. 2018;8(2): e020478. 10.1136/bmjopen-2017-020478. 29455171 10.1136/bmjopen-2017-020478 PMC5855250

[118] Cuzzilla R, Cowan FM, Rogerson S, et al. Relationships between early postnatal cranial ultrasonography linear measures and neurodevelopment at 2 years in infants born at <30 weeks’ gestational age without major brain injury. Arch Dis Child Fetal Neonatal Ed. Sep2023;108(5):511–6. 10.1136/archdischild-2022-324660. 36958812 10.1136/archdischild-2022-324660

[119] Thomas AR, Lacadie C, Vohr B, Ment LR, Scheinost D. Fine motor skill mediates visual memory ability with microstructural neuro-correlates in cerebellar peduncles in prematurely born adolescents. Cereb Cortex. 2017;27(1):322–9. 10.1093/cercor/bhw415. 28108493 10.1093/cercor/bhw415 PMC5939198

[120] Stipdonk LW, Franken MJP, Dudink J. Language outcome related to brain structures in school-aged preterm children: A systematic review. PLoS ONE. 2018;13(6): e0196607. 10.1371/journal.pone.0196607. 29864120 10.1371/journal.pone.0196607 PMC5986152

[121] Stipdonk LW, Boumeester M, Pieterman KJ, et al. Cerebellar volumes and language functions in school-aged children born very preterm. Pediatr Res. 2021;90(4):853–60. 10.1038/s41390-020-01327-z. 33469182 10.1038/s41390-020-01327-z

[122] Agrawal S, Rao SC, Bulsara MK, Patole SK. Prevalence of autism spectrum disorder in preterm infants: a meta-analysis. Pediatrics. 2018;142(3). 10.1542/peds.2018-0134. 10.1542/peds.2018-0134 30076190

[123] D’Mello AM, Crocetti D, Mostofsky SH, Stoodley CJ. Cerebellar gray matter and lobular volumes correlate with core autism symptoms. Neuroimage Clin. 2015;7:631–9. 10.1016/j.nicl.2015.02.007. 25844317 10.1016/j.nicl.2015.02.007 PMC4375648

[124] Wang SS, Kloth AD, Badura A. The cerebellum, sensitive periods, and autism. Neuron. 2014;83(3):518–32. 10.1016/j.neuron.2014.07.016. 25102558 10.1016/j.neuron.2014.07.016 PMC4135479

[125] Hadaya L, Vanes L, Karolis V, et al. Distinct neurodevelopmental trajectories in groups of very preterm children screening positively for autism spectrum conditions. J Autism Dev Disord. 2024;54(1):256–69. 10.1007/s10803-022-05789-4. 36273367 10.1007/s10803-022-05789-4 PMC10791910

[126] Tran L, Huening BM, Kaiser O, et al. Cerebellar-dependent associative learning is impaired in very preterm born children and young adults. Sci Rep. 2017;7(1):18028. 10.1038/s41598-017-18316-8. 29269751 10.1038/s41598-017-18316-8 PMC5740078

[127] Lawrence EJ, Froudist-Walsh S, Neilan R, et al. Motor fMRI and cortical grey matter volume in adults born very preterm. Dev Cogn Neurosci. 2014;10:1–9. 10.1016/j.dcn.2014.06.002. 25016248 10.1016/j.dcn.2014.06.002 PMC4256062

